# Expression of genes of the Pho regulon is altered in *Streptomyces coelicolor*

**DOI:** 10.1038/s41598-020-65087-w

**Published:** 2020-05-22

**Authors:** Aaron Millan-Oropeza, Céline Henry, Clara Lejeune, Michelle David, Marie-Joelle Virolle

**Affiliations:** 1 Université Paris-Saclay, CEA, CNRS, Institute for Integrative Biology of the Cell (I2BC), 91198 Gif-sur-Yvette, France; 2 PAPPSO, Micalis Institute, INRAE, AgroParisTech, Université Paris-Saclay, Jouy-en-Josas, France

**Keywords:** Applied microbiology, Proteomics, Data mining

## Abstract

Most currently used antibiotics originate from Streptomycetes and phosphate limitation is an important trigger of their biosynthesis. Understanding the molecular processes underpinning such regulation is of crucial importance to exploit the great metabolic diversity of these bacteria and get a better understanding of the role of these molecules in the physiology of the producing bacteria. To contribute to this field, a comparative proteomic analysis of two closely related model strains, *Streptomyces lividans* and *Streptomyces coelicolor* was carried out. These strains possess identical biosynthetic pathways directing the synthesis of three well-characterized antibiotics (CDA, RED and ACT) but only *S. coelicolor* expresses them at a high level. Previous studies established that the antibiotic producer, *S. coelicolor*, is characterized by an oxidative metabolism and a reduced triacylglycerol content compared to the none producer, *S. lividans*, characterized by a glycolytic metabolism. Our proteomic data support these findings and reveal that these drastically different metabolic features could, at least in part, due to the weaker abundance of proteins of the two component system PhoR/PhoP in *S. coelicolor* compared to *S. lividans*. In condition of phosphate limitation, PhoR/PhoP is known to control positively and negatively, respectively, phosphate and nitrogen assimilation and our study revealed that it might also control the expression of some genes of central carbon metabolism. The tuning down of the regulatory role of PhoR/PhoP in *S. coelicolor* is thus expected to be correlated with low and high phosphate and nitrogen availability, respectively and with changes in central carbon metabolic features. These changes are likely to be responsible for the observed differences between *S. coelicolor* and *S. lividans* concerning energetic metabolism, triacylglycerol biosynthesis and antibiotic production. Furthermore, a novel view of the contribution of the bio-active molecules produced in this context, to the regulation of the energetic metabolism of the producing bacteria, is proposed and discussed.

## Introduction

The *Streptomyces* genus is of crucial importance for mankind since it produces more than two thirds of life-saving antibiotics^1,2^. Consequently, worldwide research efforts have been devoted to a better understanding of the regulation of antibiotic biosynthesis^3,4^ to enhance the production of these important molecules and discover novel antibiotics^5^. The regulation of antibiotic biosynthesis is extremely complex as testified by the abundant literature of the field and by recent large-scale transposition mutagenesis studies revealing that the inactivation of several hundred of genes had a positive or a negative impact on the biosynthesis of a single antibiotic (RED or ACT) in *Streptomyces coelicolor* (*SC*)^6,7^. Therefore, despite numerous important contributions^8^, a global understanding of the complex regulation of antibiotics biosynthesis in *Streptomyces*, its links with primary metabolism and the role of these molecules in the physiology of the producing bacteria, is still incomplete.

The two closely related model strains, *Streptomyces coelicolor* (*SC*)^9,10^ and *Streptomyces lividans* (*SL*)^11^ have been extensively studied to address these questions. These strains, despite their phylogenetic proximity (93% of the genes present in one strain have orthologs in the other strain) have drastically different abilities to produce three well characterized bioactive secondary metabolites, the peptide Calcium Dependent Antibiotic (CDA), the hybrid peptide-polyketide red antibiotic undecylprodigiosin (RED) and the blue polyketide antibiotic, actinorhodin (ACT). Notably, these two strains also show a different ability to accumulate storage lipids of the TriAcylGlycerolcerol (TAG) family upon growth on the classical R2YE solid medium containing glucose as main carbon source^12–14^. On this medium, the weak antibiotic producer, *SL*, accumulates TAG but exclusively in conditions of phosphate (P) limitation, as most micro-organisms^15,16^. In contrast, the TAG content of *SC* remains at a low level throughout growth in phosphate limitation or proficiency^14^. This suggested the existence of a reverse correlation between the ability to accumulate TAG and that to synthesize antibiotics. However, we noticed that when *SL* and *SC* were grown on the same solid R2YE medium but with glycerol instead of glucose, as main carbon source, *SC*, while still producing antibiotics, was able to accumulate TAG, although to a lower level than *SL* (this study). In order to achieve a better understanding of the metabolic features underpinning the contrasted biosynthetic abilities of these two strains in relation to the nature of the carbon source used for growth (glucose or glycerol), a quantitative shotgun label-free comparative proteomic analysis was conducted.

In this study, the number of proteins showing significant differences in their abundance between *SC* and *SL* was 3 fold higher (1040 versus 360) than in our previous study carried out in liquid cultures in micro-aerobiosis^17^. This allowed a much better understanding of the metabolic differences between the two strains and revealed the unexpected significantly lower abundance, in *SC* compared to *SL*, of the sensor kinase PhoR and the response regulator PhoP. This two-component system is known to control positively and negatively, respectively, phosphate (Pi)^18–20^ and nitrogen (N) assimilation^21–23^. The low abundance of PhoR/PhoP in *SC* resulted in the tuning down of its regulatory role and thus in a lower abundance of proteins involved in Pi scavenging and uptake in *SC* than in *SL*, leading to a severe Pi limitation in *SC*. In contrast, the relieve of the repressing effect that PhoP exerts on N assimilation is expected to lead to higher N availability in *SC* compared to *SL*. Furthermore, our study also revealed putative novel PhoP regulatory targets involved in carbon as well as iron metabolism. The differential expression of the numerous Pho targets in the two strains contributed to their contrasting metabolic features. Indeed, *SL* is characterized by a glycolytic metabolism allowing TAG accumulation whereas *SC* is characterized by an oxidative metabolism generating abundant ATP and oxidative stress^14,17^. A novel view of the role played by the produced antibiotics in the regulation of the energetic metabolism of the producing bacteria in condition of Pi scarcity is proposed and discussed.

## Results

### Bacterial growth and biochemical analysis

In this study the classical solid R2YE medium with no phosphate added (condition of Pi limitation) was used to cultivate *SC* and *SL*. This medium contains 1 mM free P but most of the phosphate is organic phosphate present in the yeast extract. Similarly, most N present in this medium resides in organic molecules (amino acids, yeast extract). Access to organic P and N thus first requires the specific induction of enzymes involved in the degradation of these molecules.

**Figure 1:**
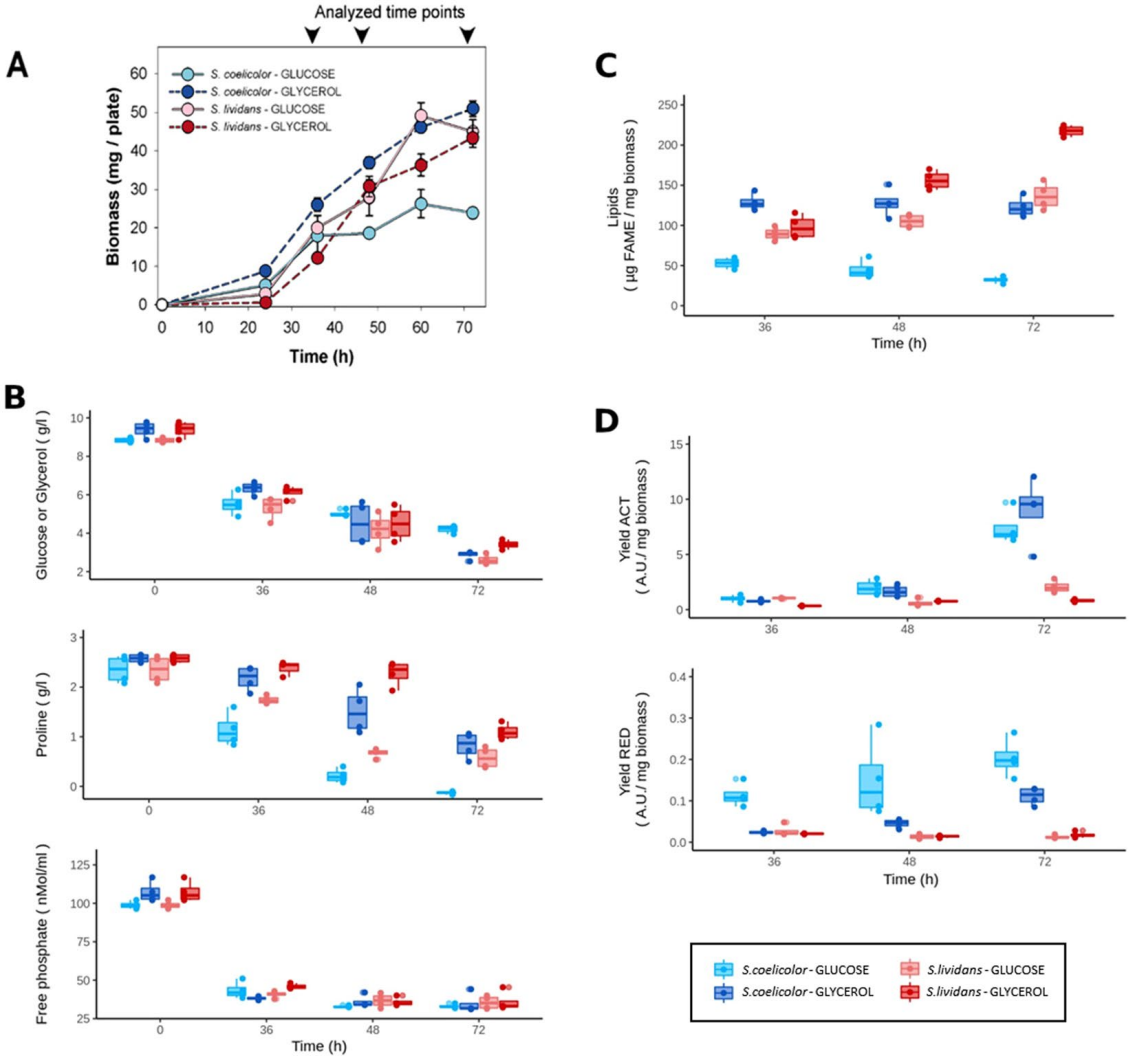
Cultures of *S. lividans* (TK24) and *S. coelicolor* (M145) grown on solid R2YE limited in phosphate (1 mM) with either glucose (50 mM) or glycerol (100 mM) as main carbon source for 36, 48 and 72 h at 28 °C. Growth curves of *S. lividans* and *S. coelicolor* grown on glucose and glycerol **(A)**. Concentration of glucose, proline and free phosphate in the growth medium **(B)**. Content in Fatty Acid Methyl Esters (FAME) **(C)**. Quantification of ACT and RED yields **(D)**.

Spores (10^6^) of *SL* and *SC* were plated on cellophane disks (porous to nutrients) deposited on the surface of plates of solid R2YE medium limited in Pi and supplemented with either glucose (50 mM) or glycerol (100 mM), as main carbon sources. Growth per plate was estimated by dry biomass weight (Fig. 1A). On glycerol, growth of the two strains was similar, however since *SL* showed an extended lag phase of 24 h, biomass yields of *SL* were slightly lower than those of *SC*. In contrast, on glucose, growth of *SC* was severely retarded compared to that of *SL* from 36 h onward. Consistently, the consumption of glucose was less active in *SC* than in *SL* (Fig. 1B) and the total Fatty Acid Methyl Esters (FAME) content of *SC* was 2 to 4 fold lower than that of *SL* (Fig. 1C). In contrast the two strains consumed glycerol at a similar rate and their FAME content was rather similar at 36 h and 48 h but at 72 h the FAME content of *SL* was 1.8 fold higher than that of *SC*. The FAME content of *SC* was 3.6 fold higher on glycerol than on glucose whereas that of *SL* was only 1.6 fold higher on glycerol compared to glucose. These data indicated that glycerol was more lipogenic than glucose for both strains. On solid (this study) as in liquid R2YE glucose medium^17^, *SC* consumed proline, the most abundant amino acid of the medium (0.3 g/l) likely to constitute the major NH_4_^+^ source, at a greater rate and finally completely compared to *SL* (Fig. 1B) whereas this difference was less pronounced on glycerol. Interestingly the absence (in glucose) or reduced (in glycerol) TAG accumulation in *SC* was correlated with ACT and RED biosynthesis (Fig. 1D). In order to obtain a better understanding of molecular basis of the different growth rate and biosynthetic abilities of the two strains in relation to the nature of the carbon sources used for growth, a deep shotgun label-free comparative analysis of the proteomes of *SL* and *SC* was carried out, at 36 h, 48 h and 72 h, in the presence of glucose or glycerol.

### Global proteome analysis

**Figure 2:**
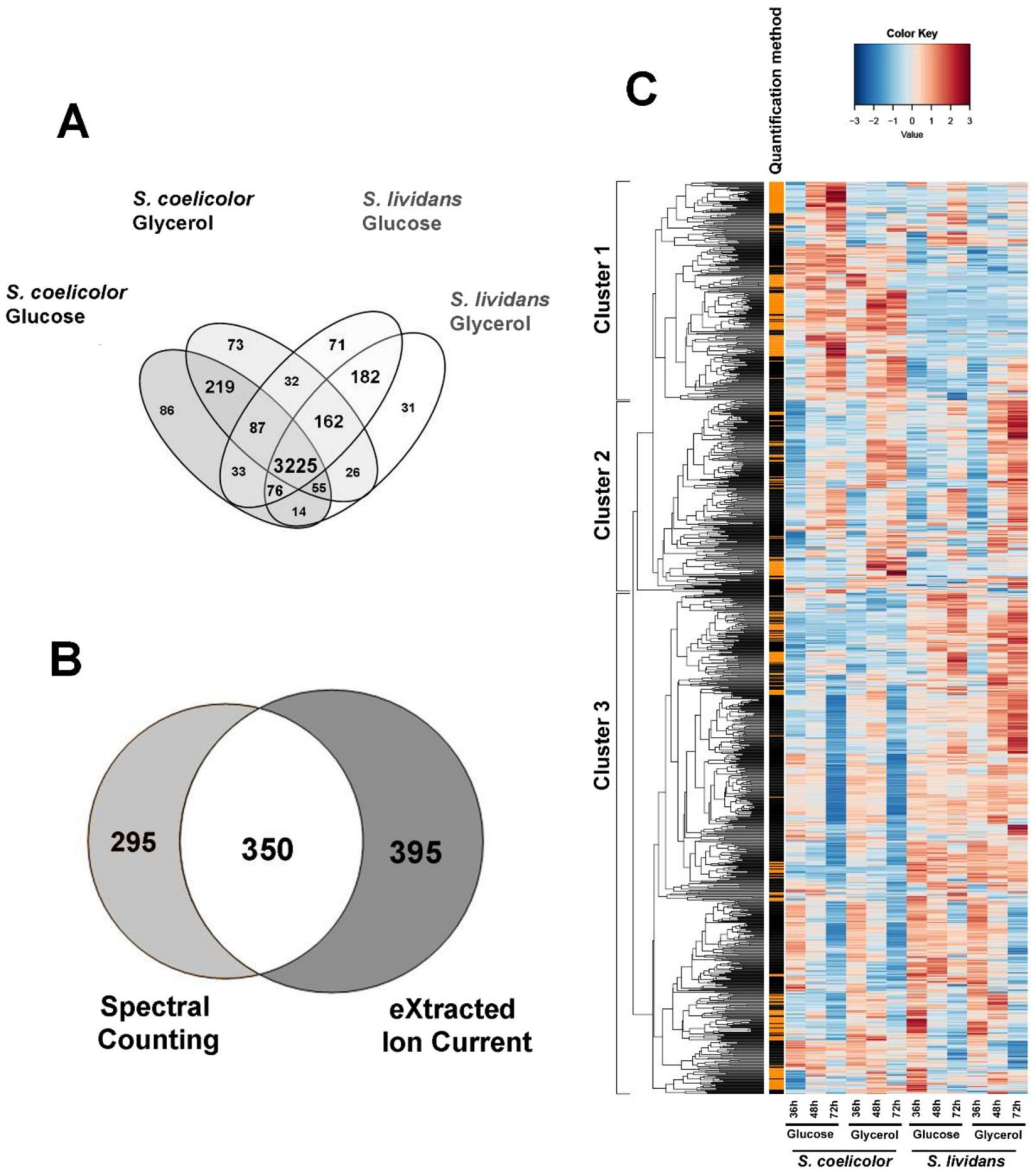
Comparative analysis of the proteomes of *S. lividans* (TK24) and *S. coelicolor* (M145) grown on R2YE medium limited in phosphate (1 mM) with either glucose or glycerol as main carbon source, for 36, 48 and 72 h. Venn diagram of the 3225 proteins identified using X!TandemPipeline showing significant abundance change according to strain, medium or time. (ANOVA, adjusted p value <0.05) **(A)**. Detection of 1040 proteins with significant abundance changes: 745 quantified by XIC, 645 by Spectral Counting and 350 by both methods **(B)**. Heatmap representation of the protein abundances estimated by the Spectral Counting and XIC approaches. Only proteins showing significant abundance change between the two strains (ANOVA, adjusted p value <0.05) are displayed. The quantification methods are displayed in the vertical bar indicating proteins quantified by Spectral Counting (orange) or XIC (black) **(C)**.

Protein quantification was performed for 42 samples out of 48. The reasons for the exclusion of these samples are detailed in Materials and Methods. A total of 4372 proteins were quantified in 42 samples by both Spectral Counts and eXtracted Ion Current (XIC) approaches, 3710 were found both in *SC* and *SL* and 378 and 284 were only detected in *SC* and *SL*, respectively (Fig. 2A, data available in File S1). These values represented approximately half of the theoretical proteome of the strains. The 1040 proteins that showed statistical abundance changes were represented in a heatmap using hierarchical clustering with Euclidean distances (Fig. 2C). Among these, 295 were detected by the Spectral Counts-based method, 395 by the XIC-based method and 350 by both methods. When a given protein showed significant abundance change by both methods, only the values obtained by XIC-based approach were considered since it is a more sensitive approach than Spectral Counts. The proteomes were clearly resolved into 3 main clusters that show similar trend on both carbon sources. Cluster 1 contains proteins generally more abundant in *SC* than in *SL*. This cluster includes most of the proteins involved in the biosynthesis of the canonical secondary metabolites, CDA, RED and ACT. Cluster 2 includes proteins with a similar expression pattern in both strains. Finally, cluster 3 is constituted by proteins more abundant in *SL* than in *SC*.

An interesting feature revealed by this study was the significantly lower (3 to 5 fold) abundance of the sensor kinase PhoR (SCO4231) and the response regulator PhoP (SCO4230) in *SC* compared to *SL*. The relative abundance of proteins known to be under the positive or negative control of PhoR/PhoP was thus examined in the two strains.

### Proteins involved in phosphate and nitrogen assimilation are respectively down- and up-regulated in *S. coelicolor*

**Figure 3:**
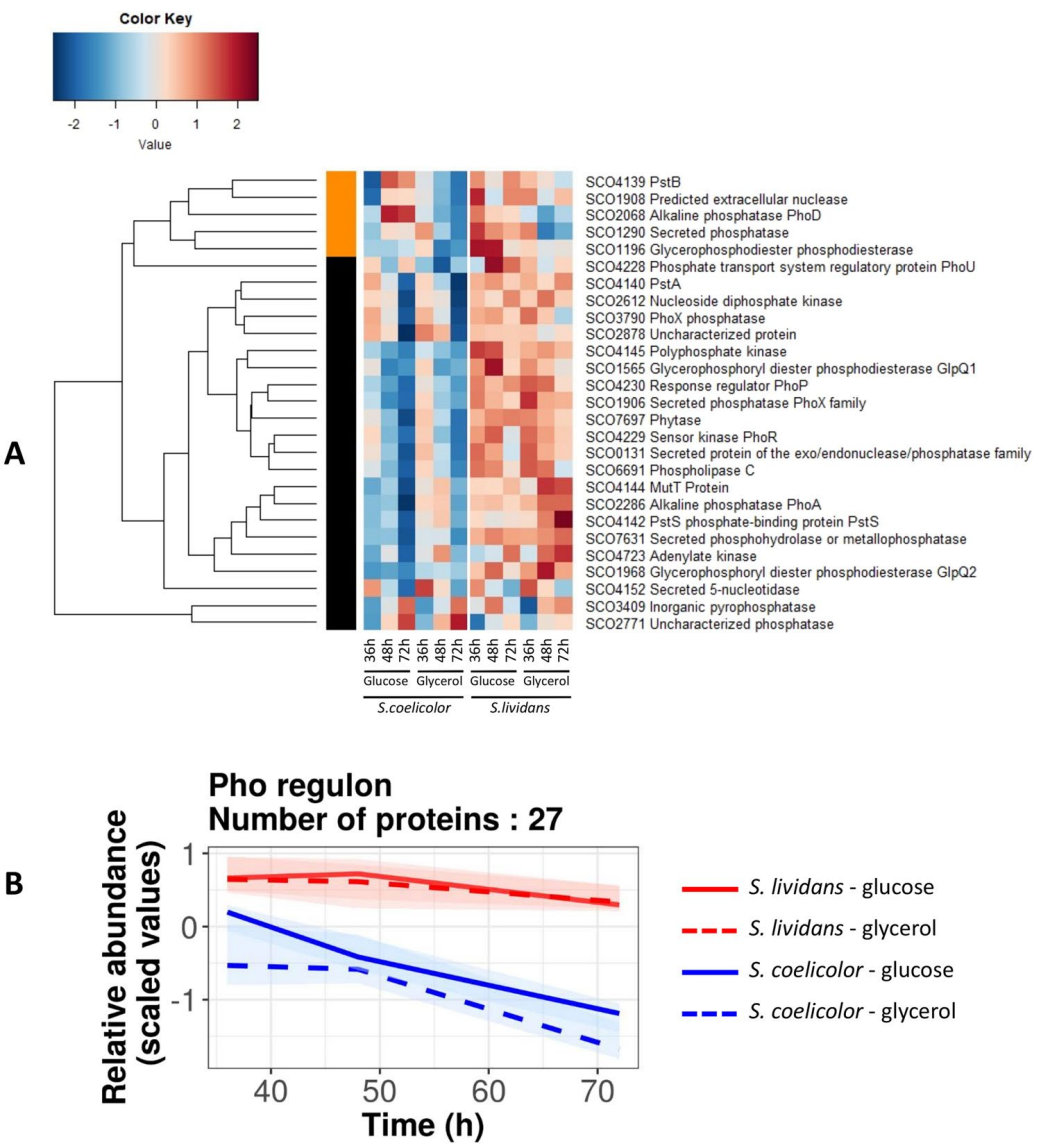
Heatmap representation of proteins of the Pho regulon belonging to phosphate metabolism with significant abundance change (ANOVA, adjusted p value <0.05) between *S. coelicolor* (M145) and *S. lividans* (TK24) with either glucose or glycerol as main carbon source, for 36, 48 and 72 h. The quantification methods are displayed in the vertical bar indicating those proteins quantified by Spectral Counting (orange) or XIC (black) **(A)**. Corresponding global trends curves **(B)**.

PhoR/PhoP is known to control positively phosphate scavenging and uptake^18–20^ as well as strategies involved in Pi saving such as the replacement of phosphate rich teichoic acids by phosphate free teichuronic acids^22^,^24^ or of phospholipids by phosphate free ornithine lipids^25^. As anticipated, the low abundance of PhoR/PhoP in *SC* was correlated with significantly lower abundance of proteins known to be positively regulated by PhoP, in *SC* compared to *SL* (Fig. 3). This was confirmed at the transcriptional level, by qRT-PCR experiments, for 8 genes known to belong to the Pho regulon (Fig. S2A). Furthermore since most of the phosphate present in R2YE is organic phosphate, its mobilization necessitates the action of various PhoP-dependent phosphatases that were also less abundant in *SC* than in *SL* (Fig. 3A).

PhoR/PhoP is also known to regulate negatively the expression of several genes involved in nitrogen (N) assimilation^21–23^ either directly or indirectly *via* its repressive effect on the expression of GlnR (SCO4159), a major positive or negative regulator of nitrogen (N) metabolism^26–29^. The expression of GlnR seems independent of N availability but its affinity for its targets was demonstrated to be modulated by phosphorylation^30^. GlnR has a pleiotropic impact on both primary and secondary cellular metabolism^31^ not only *via* its impact on the expression of genes of N metabolism but also through the positive control it exerts on the expression of lysine deacetylases that modulate the activity of numerous proteins *via* removal of post-translational acetylations^32^. In our study, the abundance of GlnR was too low to reveal significant difference between the two strains and most proteins belonging to the GlnR regulon had a similar abundance in the two strains. However, one notes the slightly higher abundance in *SC* of proteins known to be positively regulated by GlnR such as the glutamine synthetases I and II (SCO2198 and SCO2210). Since glutamine is the first amino acid to be synthesized from glutamate in conditions of high ammonium (NH_4_^+^) concentration, this indicated higher glutamate and NH_4_^+^ availability (likely originating from proline degradation) in *SC* compared to *SL*. Furthermore, the high abundance of SCO7428, a protein belonging to the NsrR regulon (File S2), involved in the conversion/detoxification of nitric oxide (NO) to nitrate^33^, of nitrite reductase (SCO0216-17) belonging to the dormancy regulon OsdR^34^ as well as of other nitrite (NirB, SCO2487) or nitrate reductases, SCO2473 and SCO4947-48 whose expression was shown to be induced in Pi limitation^35^ was noteworthy in *SC* (File S2). This suggested the existence of NO stress detected by the WhiB-like proteins in *SC*^36^. Such stress might contribute to the slow growth rate of *SC* and its entry into a dormancy state as reported for *Mycobacterium*^37^. The predicted high N availability in *SC* is proposed to lead to inhibition of TAG biosynthesis as in other micro-organisms^38–40^.

The relationships between P and N metabolism in Streptomycetes have been established in previous studies and our data are consistent with what was reported^23^. However, the proper functioning of cellular metabolism requires the coordinated regulation of C, P, N and energetic metabolisms. In consequence, the differences in the abundance of proteins belonging to central carbon metabolism in *SC* and *SL* were examined.

### Enzymes of  Glycolysis, Pentose Phosphate Pathway, Pyruvate Deshydrogenase complex and over-flow metabolism were more abundant in *S. lividans* than in *S. coelicolor*, except SCO7511 and SCO4209

Among the 140 proteins of carbon metabolism that showed significant abundance variation between strains and/or nature of the carbon sources, 72 proteins belong to conserved pathways of central carbon metabolism.

**Figure 4:**
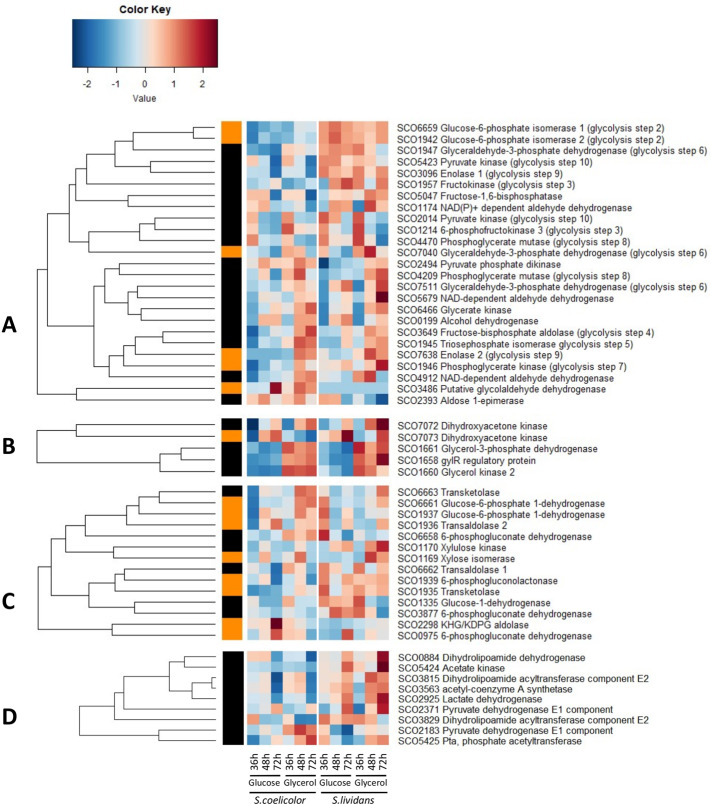
Independently generated heatmap representations of proteins belonging to central carbon metabolism pathways with significant abundance change (ANOVA, adjusted p value < 0.05) between *S. coelicolor* (M145) and *S. lividans* (TK24) with either glucose or glycerol as main carbon source, for 36, 48 and 72 h. Proteins belonging to glycolysis/gluconeogenesis **(A)**; glycerol metabolism **(B)**; Pentose Phosphate Pathway **(C)**; AcetylCoA generation and over-flow metabolism **(D)**. Protein identifiers are indicated as SCO numbers for both strains and by predicted functions. The quantification methods are displayed in the vertical bar indicating proteins quantified by Spectral Counting (orange) or XIC (black).

On glucose, enzymes catalyzing the first four glycolytic steps resulting in the production of the phosphorylated trioses GAP and DHAP were clearly more abundant in *SL* than in *SC* (Fig. 4A). On glucose, these trioses whose inter-conversion is catalyzed by the triose phosphate isomerase SCO1945 (TPI, step 5) can then be converted into glycerol 3 P (Glycerol3P) by glycerol 3P dehydrogenases whereas on glycerol, Gly3P results from the phosphorylation of glycerol by an ATP dependent glycerol kinase (SCO1660)^41^ (Fig. 4B). Gly3P is a necessary precursor of PhosphoLipids (PL) and/or TAG biosynthesis. Step 6 is putatively catalyzed by 3 different NAD+-dependent glyceraldehyde-3-phosphate dehydrogenases (GAPDH SCO7511, SCO1947 and SCO7040), step 7 by the phosphoglycerate kinase (PGK, SCO1946), step 8 by two different phosphoglycerate mutases (PGM, SCO4209 and SCO4470), step 9 by two different enolases (ENO, SCO3096 and SCO7638) and step 10 by the pyruvate kinase (PYK, SCO2014)^42^. Interestingly, the Fructose bis-phosphate aldolase (SCO3649, step 4), the TPI SCO1945, the pyruvate phosphate dikinase SCO2494 and the fructose 1, 6 bisphosphatase SCO5047 were more abundant on glycerol than on glucose in both strains at 48 h and 72 h. The abundance of these enzymes catalyzing the glycolytic steps upstream of the entry point of glycerol into glycolysis, suggests that they contribute to gluconeogenesis from glycerol (Fig. 4A). The GADPH SCO7040, the PGK SCO1946-47, the PGM SCO4470 and the ENO SCO3096 were more abundant in *SL* than in *SC* on both carbon sources throughout growth as enzymes of the upper part of glycolysis (Fig. 4A). Interestingly the ENO SCO7638 and the PYK SCO2014 were expressed at a far higher level (up to 7 fold) than the other glycolytic enzymes in *SL* (Fig. 4A). In contrast, at 36 h and/or 48 h, the GAPDH SCO7511 and PGM SCO4209 were 5.6 and 1.9 fold and 2.6 and 1.7 fold more abundant in *SC* than in *SL* on glycerol and glucose, respectively.

Enzymes constituting the PPP pathway (Fig. 4C), the pyruvate dehydrogenase complex (PDH) or contributing to the generation and re-utilization of acetate (over-flow metabolism) were all more abundant in *SL* than in *SC* on both carbon sources throughout growth or at different time points (Fig. 4D). The higher abundance of these enzymes in *SL* compared to *SC* is consistent with its more active glucose uptake, higher glycolytic activity and ability to accumulate TAG, a process requiring abundant NADPH synthesized by the PPP.

### PhoP likely regulates the expression of some genes encoding glycolytic enzymes

We considered the possibility that the higher abundance in *SC* than in *SL* of the GAPDH SCO7511 and the PGM SCO4209 or the lower abundance in *SC* than in *SL* of the ENO SCO7638 and PYK SCO2014 (Fig. S6A) could be due to the direct/indirect negative or positive control of the expression of their encoding genes by PhoP. In order to test this hypothesis the level of expression of these genes was assessed in qRT-PCR with RNA prepared from *SL* and its *phoP* mutant^43^ as well as from *SC* for comparison (Fig. S6B). We first noticed that protein and transcript abundance were correlated in *SL* and *SC* for *sco2014* and *sco7638* but not for *sco7511* and *sco4209*. This lack of correlation between transcriptome and proteome is not unusual and has been frequently reported^44^. The transcriptional start site of these genes was positioned according to Jeong *et al*.^45^ and Pho boxes^22,24,46^ were searched in their promoter regions (Fig. S6C).

qRT-PCR data indicated that the level of expression of *sco7511* and *sco2014* was higher in the wild type strain of *SL* than in its *phoP* mutant suggesting that the expression of these genes was under the positive control of PhoP. Inspection of the promoter region of *sco2014* did not reveal the presence of a canonical Pho box (constituted by 2 direct repeats) but a half Pho box and numerous TCA repeats were found upstream of the −35 promoter region of *sco2014* (Fig. S6C2) and could be the siege of a positive regulation by PhoP. Inspection of the promoter region of *sco7511* did not reveal either the presence of a canonical Pho box (constituted by 2 direct repeats) but a half Pho box was detected just upstream of the −35 sequence and might play the role of activator site.

In contrast, the level of expression of *sco4209* and *sco7638* was slightly higher in the *phoP* mutant than in the wild type strain suggesting that the expression of these genes might be under the negative control of PhoP. Inspection of the promoter region of *sco4209* revealed the presence of putative Pho box located downstream of its −10 promoter region (Fig. S6C3) and that of *sco7638* revealed the presence of two putative Pho boxes, one overlapping the −35 promoter region (position of repressor site) and the other one found just upstream the ATG start codon (position of road block) (Fig. S6B4). These positions are consistent with the repression of *sco4209* and *sco7638* expression by PhoP. However, since the level of expression of these genes was not significantly higher in the *phoP* mutant than in *SL* we cannot exclude that other regulators besides PhoP contribute to their regulation.

Even if the difference of level of expression of the tested genes (especially that of *sco7511* and *sco2014*) between the wild type and the *phoP* mutant of *SL* argues in favor of their regulation by PhoP, the functionality of the putative Pho-boxes found in the promoter region of these genes should be confirmed experimentally by site-directed mutagenesis and EMSA. Furthermore most of the genes tested (except *sco2014*) do not show similar regulatory features in the *phoP* mutant of *SL* and in *SC* pointing out that *SC* should not be considered as a simple *phoP* mutant.

### Enzymes of the Tricarboxylic acid cycle and those involved in the degradation of amino acids generating reduced co-factors were up-regulated in *S. coelicolor*

**Figure 5:**
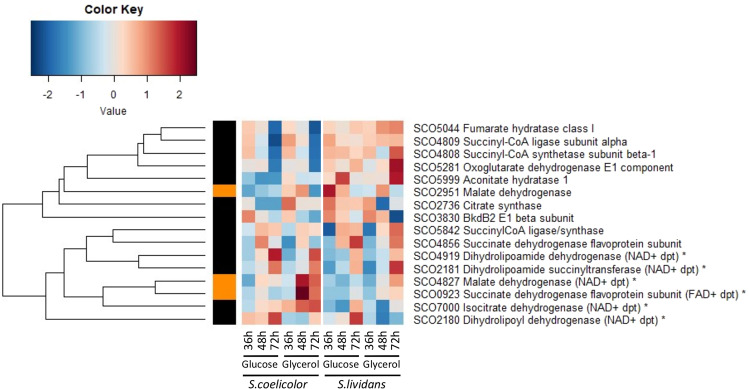
Heatmap representation of proteins belonging to the Tricarboxylic Acid Cycle with significant abundance change (ANOVA, adjusted p value<0.05) between *S. coelicolor* (M145) and *S. lividans* (TK24) with either glucose or glycerol as main carbon source, for 36, 48 and 72 h. Protein identifiers are indicated as SCO numbers for both strains and by predicted functions. Enzymes with asterisks are catalyzing a reaction generating a reduced co-factor. The quantification methods are displayed in the vertical bar indicating proteins quantified by Spectral Counting (orange) or XIC (black).

Most enzymes of the Tricarboxylic acid cycle (TCA) and notably those of the upper part of the TCA, the citrate synthase SCO2736 and the aconitase SCO5999 as well as the NADP+ dependent malic enzyme SCO2951^47^, were more abundant in *SL* than in *SC* throughout growth on glucose and glycerol, to a lesser extent. This is consistent with the more active growth and thus higher anabolic activity of *SL* (Fig. 5) as well as with its high TAG content since malic enzymes are thought to play a positive role (likely *via* NADPH generation) in lipid accumulation^48,49^. In contrast, enzymes of the lower part of the TCA, that generate reduced co-factors, were 2 to 7 fold more abundant in *SC* than in *SL* on glycerol and on glucose, to a lesser extent at 48 h and 72 h (Fig. 5). These include, the NAD+-dependent isocitrate dehydrogenase SCO7000, as well as SCO2180, SCO2181 and SCO4919 putatively constituting the NAD+ dependent α-ketoglutarate dehydrogenase complex converting α-ketoglutarate into succinate; SCO0923 thought to be part of the FAD+-dependent succinate dehydrogenase complex converting succinate into fumarate that also constitutes the complex II of the respiratory chain and SCO4827, a NAD+ dependent malate dehydrogenase converting malate into oxaloacetate. This indicated that in *SC* the TCA is mainly fueled by α–ketoglutarate resulting from proline degradation.

Furthermore, interestingly, 9 among the 36 proteins involved in amino acid degradation, showing significant abundance variation between strains and/or nature of the carbon sources, were more abundant in *SC* than in *SL* but mainly on glucose (Fig. S5). These enzymes catalyzed reactions generating reduced co-factors, including 4 enzymes involved proline catabolism (Fig. S5). The unexpected up-regulation of these enzymes in *SC* will be commented in the discussion.

### Proteins of the respiratory chain and of the ATP synthase complex were more abundant in *S. lividans* than in *S. coelicolor*

Fifteen of the 17 proteins annotated as involved in the respiratory chain including ATP synthase were clearly more abundant in *SL* than in *SC* at all time points (Fig. S7). Twelve of these 17 proteins are part of the Rex regulon and are negatively regulated by Rex (http://regprecise.lbl.gov/RegPrecise/regulon.jsp?regulon_id=63393, Fig. S7). Rex senses changes in the cellular NADH/NAD+ redox balance and an excess of NADH is thought to hinder Rex binding to its target sites. High NADH/NAD+ ratios thus result in alleviation of Rex mediated repression^50^. Such regulation adjusts the abundance of proteins of the respiratory chain to the amount of NADH generated by cellular metabolism. Eleven out of the 12 proteins belonging to the Rex regulon were more abundant in *SL* than in *SC* at all time points. Only one protein of the Rex regulon, SCO4568, was more abundant in *SC* throughout growth on glucose. This protein is the sub-unit G of the NADH dehydrogenase constituted by 14 sub-units (A to N) encoded by the large operon SCO4562-75. The NADH dehydrogenase and the succinate dehydrogenase complex mentioned above constitute entry points for electrons into the respiratory chain and as such might be regulated differently than the other enzymes of the respiratory chain belonging to the Rex regulon. The lower abundance of proteins of the respiratory chain in *SC* could be seen as contradictory with the up-regulation of enzymes generating reduced co-factors and with the high intracellular ATP concentration of this strain^14^. However, it is possible that, in *SC*, high ATP levels exert a negative feedback regulatory control on the expression of enzymes of the respiratory chain.

### Lipid metabolism is more active in *S. lividans* than in *S.coelicolor*

A major difference between *SC* and *SL* is their Fatty Acid Methyl Ester (FAME) content thought to be mostly due to their different TAG content^13,14^. Comparative analysis of 38 proteins annotated as involved in lipid metabolism showed a significant change in abundance between the two strains. Among them, 19 were annotated as involved in fatty acid/lipid biosynthesis and 19 in lipid degradation.

**Figure 6:**
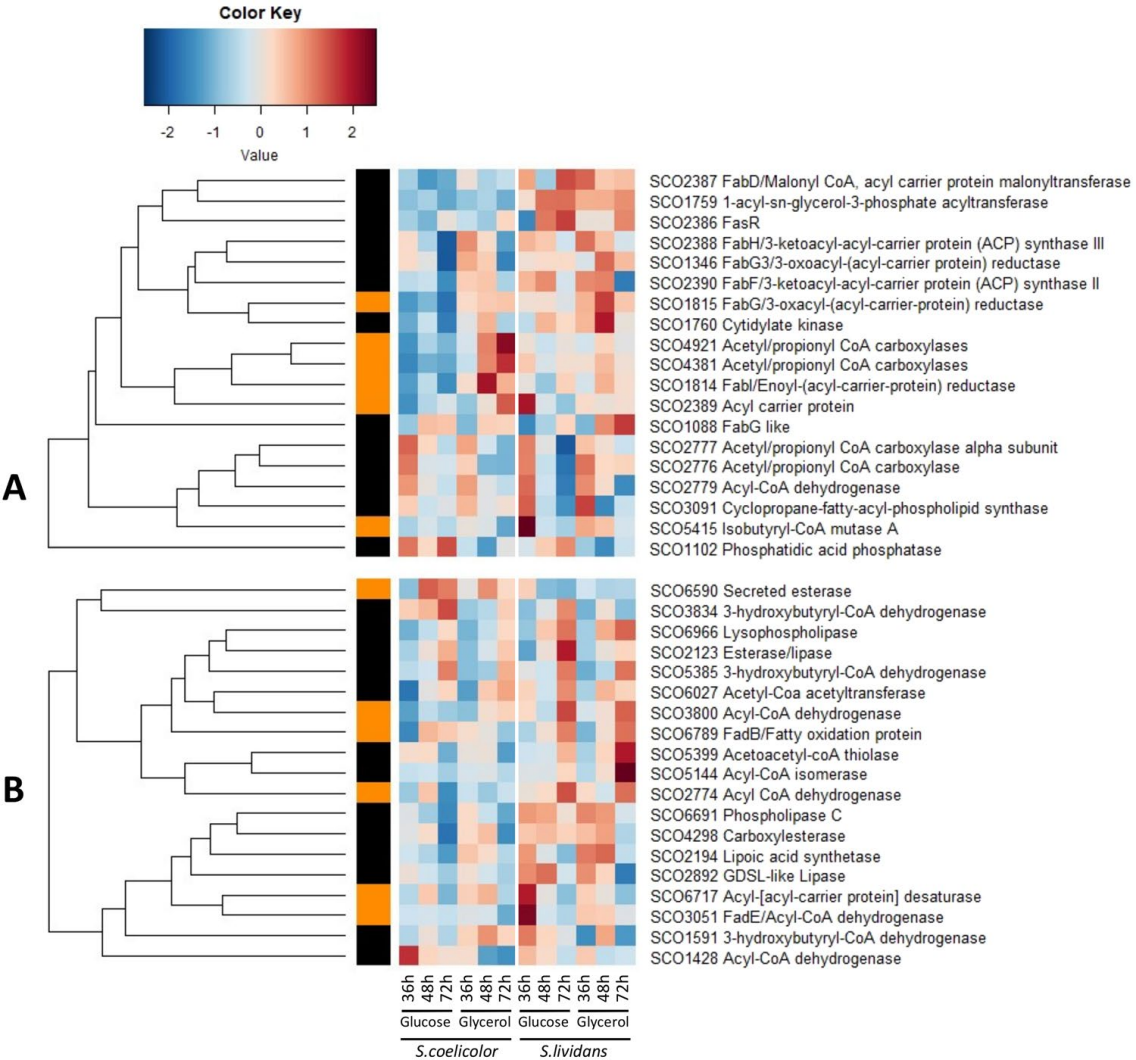
Independently generated heatmap representations of proteins belonging to lipid biosynthetic **(A)** and degradative **(B)** pathways with significant abundance change (ANOVA, adjusted p value<0.05) between *S. coelicolor* (M145) and *S. lividans* (TK24) with either glucose or glycerol as main carbon source, for 36, 48 and 72 h. Protein identifiers are indicated as SCO numbers for both strains and by predicted functions. The quantification methods are displayed in the vertical bar indicating proteins quantified by Spectral Counting (orange) or XIC (black).

This study revealed the slightly greater abundance (less than 2 fold) of most enzymes involved in fatty acid biosynthesis in *SL* compared to *SC*, especially on glucose (1.4 to 2 fold) (Fig. 6A). These include the regulator FasR (SCO2386)^51^ and most of the proteins under its positive control. Interestingly the Acyl Carrier Protein (ACP/SCO2389) that also belongs to the FasR operon was up to 12 fold more abundant in *SL* than in *SC* on glucose, at 36 h. ACPs are critical actors in fatty acid metabolism since they covalently link all fatty acyl intermediates that are in the process of being elongated (or degraded). The greater abundance of this protein compared to that of the other proteins of the Fab operon in *SL* suggested that its expression might be under the positive control of another regulator besides FasR (Fig. S6A5). This other regulator could possibly be PhoP. Consistently, the expression of this gene was significantly lower in the *phoP* mutant than in the wild type strain of *SL* (Fig. S6B5) and a putative Pho box, constituted of 2 Direct Repeats (DR) of the canonical motif, was found just upstream of the −35 region of *sco2389*, in position of activator site (Fig. S6C5). The functionality of this putative Pho-box has to be confirmed experimentally but since PL biosynthesis mobilizes an important fraction of available Pi, phosphate and lipid metabolism ought to be linked and PL biosynthesis cannot take place if sufficient Pi is not available. Furthermore, the phosphatidic acid phosphatase SCO1102 was unexpectedly far less abundant on glycerol than on glucose in both strains. This protein dephosphorylates phosphatidic acid (PA) to DiAcylGlycerol (DAG), the precursor of TAG. The low abundance of SCO1102 on glycerol could explain the lower TAG content of *SL* on glycerol than on glucose even though the availability of Glycerol3P, the precursor of PA, is expected to be higher on glycerol than on glucose.

A given lipid content results from the equilibrium between synthetic and degradation processes. Most proteins annotated as involved in fatty acid degradation fall into two classes, those expressed early (36 and 48 h), the putative lipases or phospholipases SCO4298, SCO2892 and SCO6691 and those expressed late (72 h), the putative lipase and lysophospholipase, SCO2123 and SCO6966, both classes being more abundant in *SL* than in *SC* with a few exceptions (Fig. 6B). Early in growth, the putative phospholipase C, SCO6691, is proposed to cleave off the polar head of membranous PL yielding DAG a precursor of TAG synthesis. The two other lipases expressed early might act as acyl transferases cleaving off acylCoA from PL and transferring them on DAG to generate TAG. In contrast, the lipases expressed late might cleaved off acylCoA from TAG and the latter would be degraded by the enzymes of the β-oxidation pathways that were more abundant in *SL* than in *SC*, mainly on glucose (Fig. 6B). This would allow the generation of acetylCoA and energy, late in growth in *SL*. Our proteomic data are consistent with the high TAG content of *SL* that results from a more active lipid biosynthesis in *SL* than in *SC*, especially on glucose.

### Stress responses of *S. lividans* and *S. coelicolor* differ

**Figure 7:**
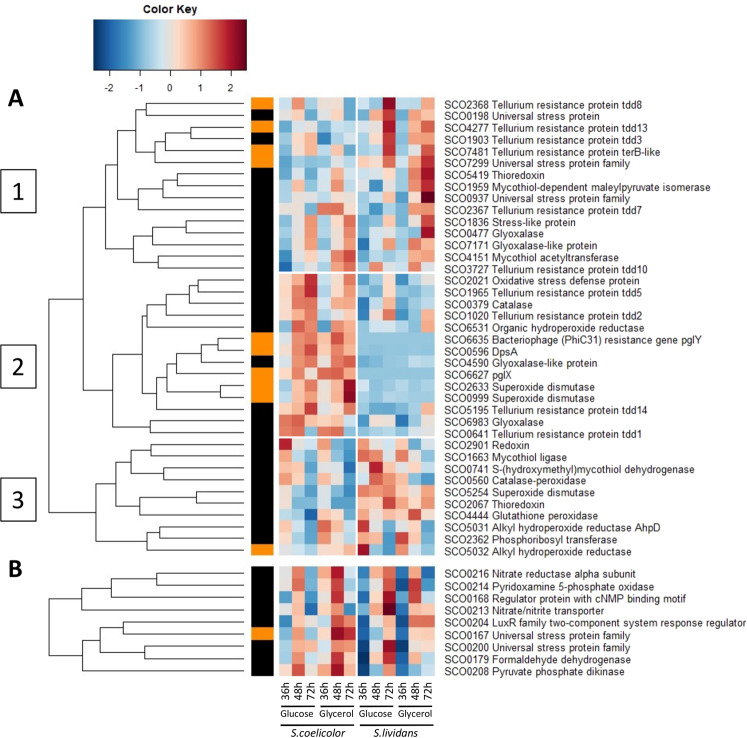
Independently generated heatmap representations of proteins involved in stress response and defense with significant abundance change (ANOVA, adjusted p value<0.05) between *S. coelicolor* (M145) and *S. lividans* (TK24) with either glucose or glycerol as main carbon source, for 36, 48 and 72 h. General stress resistance proteins (clusters 1 to 3) **(A)**. Proteins belonging to the OsdR regulon **(B)**. Protein identifiers are indicated as SCO numbers for both strains and by predicted functions. The quantification methods are displayed in the vertical bar indicating proteins quantified by Spectral Counting (orange) or XIC (black).

The abundance of 48 proteins involved in stress and defense responses showed a significant variation according to strain, medium and/or time. These proteins fall into 3 clusters (Fig. 7A).

Cluster 1 includes proteins more abundant in *SL* than in *SC* but mainly at late time points and on glycerol. These include the glyoxalases SCO7171 and SCO0477. These enzymes are thought to convert methylglyoxal (MG) into lactate. MG is a toxic by-product of glycolysis that contributes to oxidative stress^52^. MG is synthetized by an MG synthase that catalyzes the elimination of phosphate from DHAP^53^, that might be generated in higher amount on glycerol than on glucose. This reaction takes place when cells are starved for Pi, which is likely to be the case but mainly late in growth in *SL*. Some proteins involved in the maintenance of the redox balance of the cell also belonged to this cluster such as proteins involved in mycothiol (MT) biosynthesis^54^ or using MT as co-factor (SCO4151, SCO1959) as well as the thioredoxin SCO5419. At last, 6 among the 9 tellurium resistance proteins (TRP) detected (SCO1903, SCO2367-68, SCO3767, SCO4277 and SCO7481) and 3 among the 5 Universal Stress Proteins (USP) detected (SCO0198, SCO0937 and SCO7299) also belong to cluster 1. The function of TRP and USP remains unclear but reports in the literature indicate that they play a role when growth slow down to improve cell survival^55,56^.

Cluster 3 includes proteins that were also more abundant in *SL* than in *SC* but mainly on glucose and at the three time points (Fig. 7A). These include proteins belonging to mycothiol metabolism (SCO0741 and SCO1663) and the SOD SCO5254 involved in the conversion of the anion superoxide into H_2_O_2_ as well as H_2_O_2_ scavengers such as the glutathione peroxidase SCO4444, the alkyl hydroperoxide reductases (SCO5031-32) and the catalase-peroxidase SCO0560. This suggested that growth on glucose generates more oxidative stress than growth on glycerol in *SL*.

Cluster 2 includes protein that were more abundant in *SC* than in *SL* on both carbon sources and throughout growth (Fig. 7A). These include two glyoxylases, SCO4590 and SCO6983. The abundance of these enzymes throughout the growth of *SC* is consistent with the severe Pi limitation characterizing this strain and thus generation of MG from DHAP. Cluster 2 also includes some proteins involved in the resistance to oxidative stress such as SCO2021, DpsA (SCO0596) that contributes to DNA protection during severe oxidative stress^57,58^, the superoxide dismutases (SOD) SCO0999 and SCO2633, the H_2_O_2_ scavengers including the catalase SCO0379 and the alkyl hydroperoxide reductases (SCO3132 and SCO6531). This indicates that *SC* suffers oxidative stress consistently with its oxidative metabolism. This cluster also includes 3 among the 9 tellurium resistance proteins (TRP) detected (SCO0641, SCO1020 and SCO1965) and 2 among the 5 the Universal Stress Proteins (USP) detected (SCO0167 and SCO0200).

**Figure 8:**
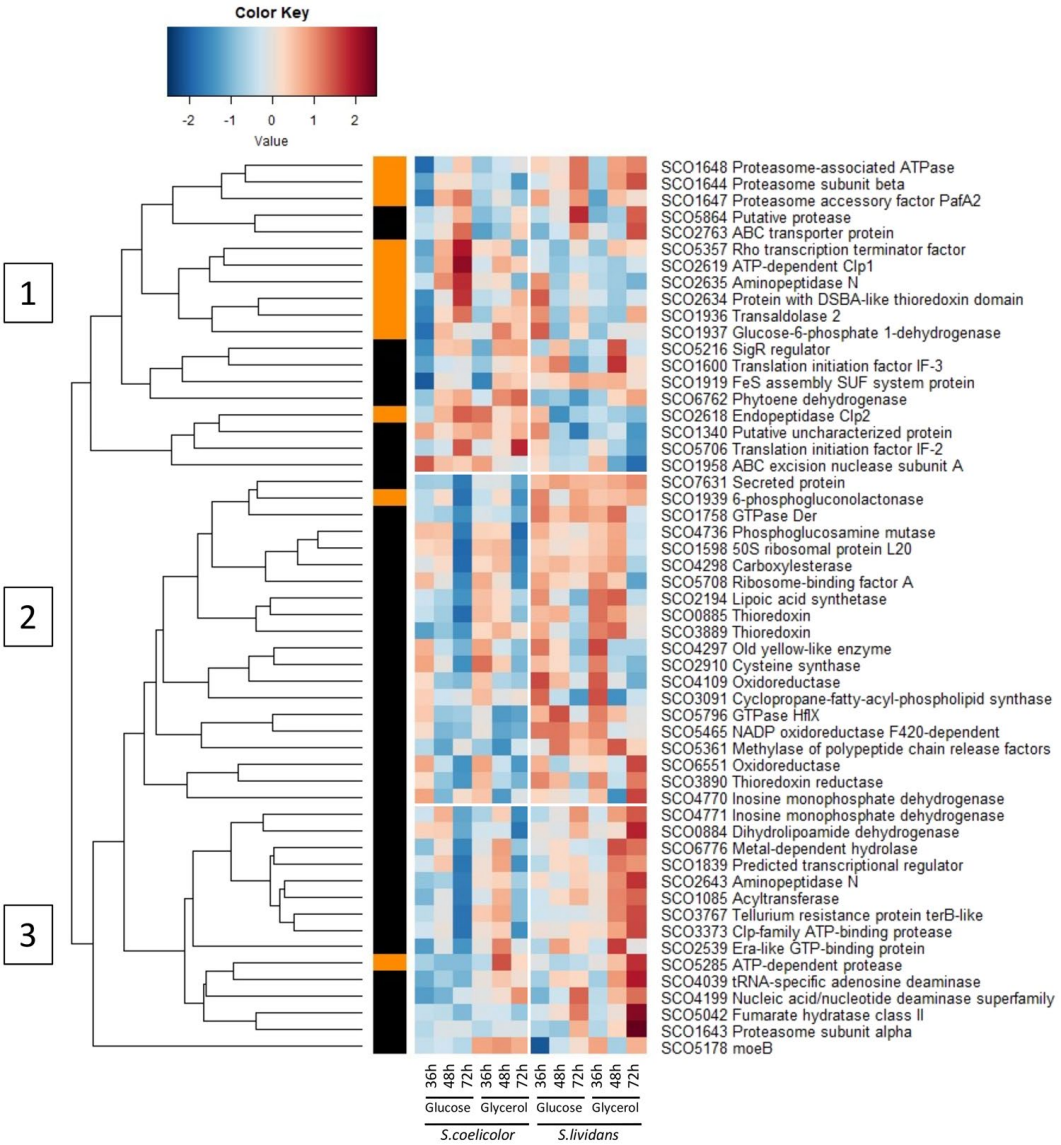
Heatmap representation of proteins belonging to the SigR regulon (clusters 1 to 3) with significant abundance change (ANOVA, adjusted p value<0.05) between *S. coelicolor* (M145) and *S. lividans* (TK24) with either glucose or glycerol as main carbon source, for 36, 48 and 72 h. Protein identifiers are indicated as SCO numbers for both strains and by predicted functions. The quantification methods are displayed in the vertical bar indicating proteins quantified by Spectral Counting (orange) or XIC (black).

Furthermore, interestingly, the anti-sigma factor antagonist SCO7324 was 30 and 10 fold more abundant in *SC* on glucose and glycerol, respectively at 72 h (File S1). SCO7324 is the first gene of a five-gene operon encoding several anti-sigma or anti-sigma-antagonists. It is related to RsbS and RsbR proteins involved in the sensing of environmental stress and the formation of the stressosome complex in *Bacillus subtilis*^59^. Its abundance indicates severe stress late in growth in *SC*. One also notes the high abundance of proteins of the dormancy regulon OsdR^34^ in *SC* (Fig. 7B). The OsdR regulon is mainly constituted by 2 large loci (SCO0161-SCO0181 and SCO0197-SCO0220) under the positive control of OsdR, the LuxR response regulator SCO0204. Some of these proteins are related to N metabolism and might be induced by NO stress as mentioned above. At last and unexpectedly 35 of the 54 proteins of the SigR regulon^60^ detected (among 77) were more abundant in *SL* than in *SC* (Fig. 8B). The SigR regulon is induced when oxidation of thiols by reactive oxygen species (ROS) results in non-native disulfide bond formation in proteins. Thioredoxins^61^ belonging (SCO0885 and SCO3889- 3890) or not (SCO2634, SCO2067, SCO2901, SCO5419) to the SigR regulon are involved in the reduction of these “illegitimate” bonds and were more abundant in *SL* throughout growth on both carbon sources. The strong and weak induction of the SigR regulon in *SL* and *SC*, respectively, was unexpected and will be commented in the discussion.

### The specific metabolic features of  *S. coelicolor*  and *S. lividans*  are correlated with the expression of specific secondary metabolites pathways

**Figure 9:**
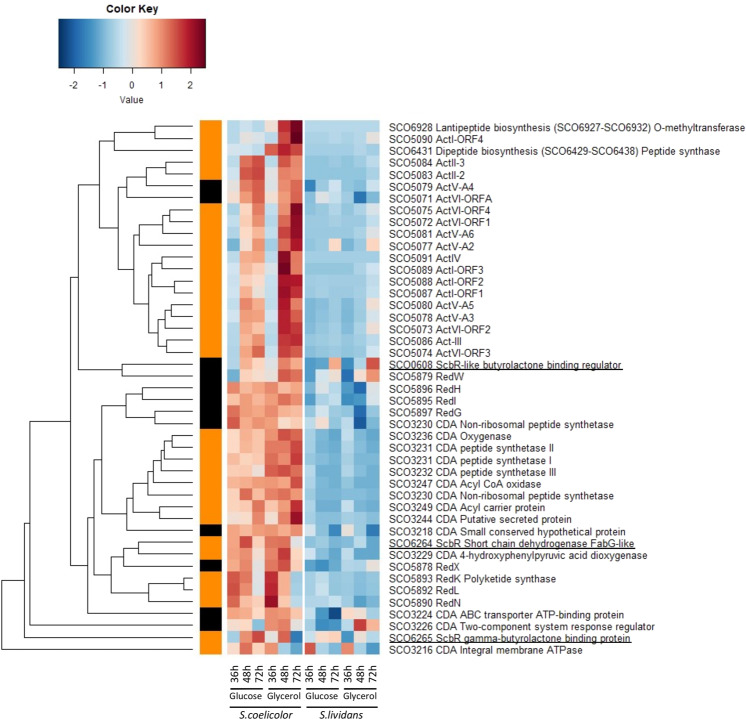
Heatmap representation of proteins belonging to Calcium Dependent Antibiotic (CDA), Red-pigmented cell-associated undecylprodigiosin (RED) and Actinorhodin (ACT) pathways with significant abundance change (ANOVA, adjusted p value<0.05) between *S. coelicolor* (M145) and *S. lividans* (TK24) with either glucose or glycerol as main carbon source, for 36, 48 and 72 h. Protein identifiers are indicated as SCO numbers for both strains and by predicted functions. ScbR proteins are underlined. The quantification methods are displayed in the vertical bar indicating proteins quantified by Spectral Counting (orange) or XIC (black).

**Figure 10:**
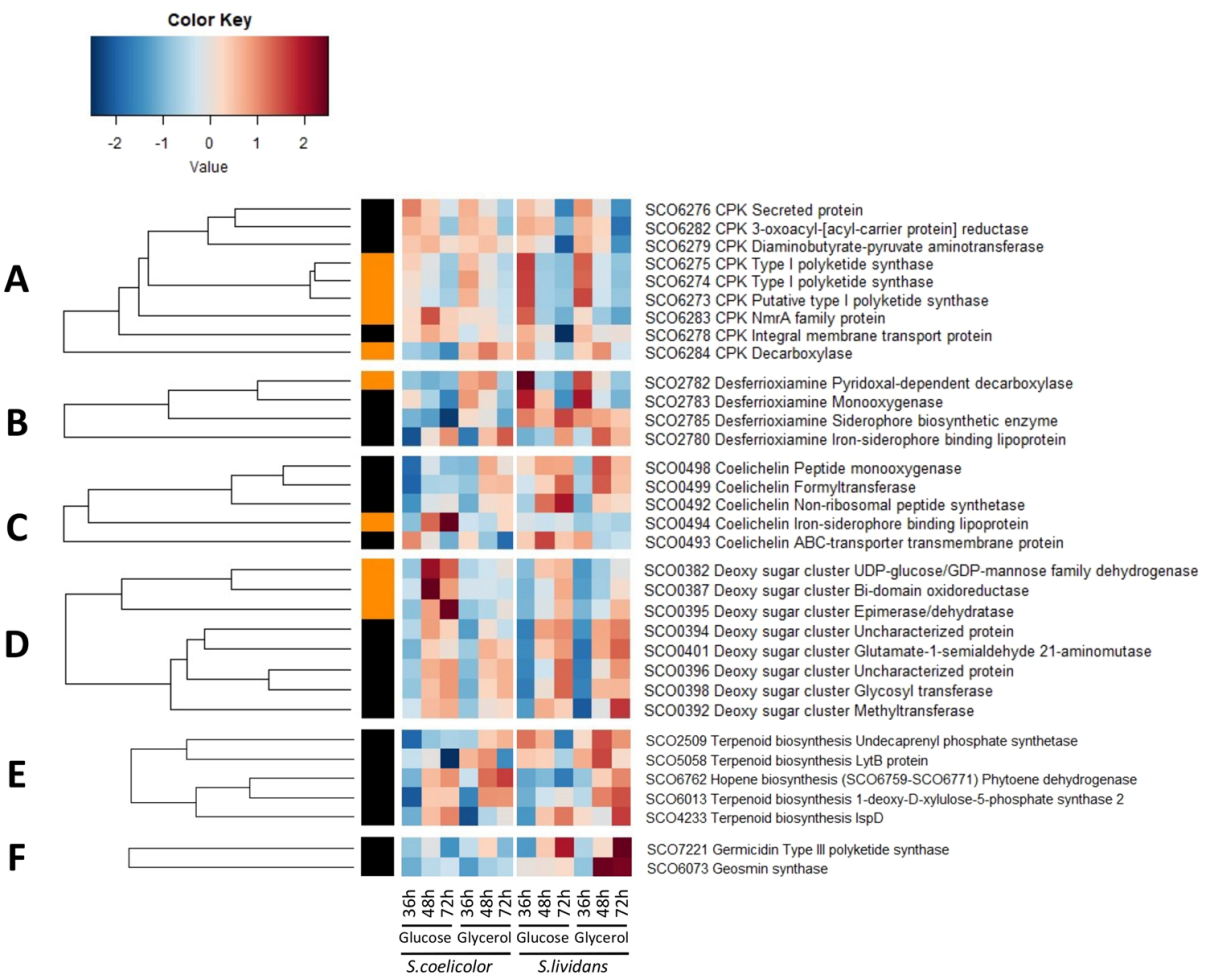
Independently generated heatmap representations of proteins belonging to others secondary metabolite pathways with significant abundance change (ANOVA, adjusted p value<0.05) between *S. coelicolor* (M145) and *S. lividans* (TK24) with either glucose or glycerol as main carbon source, for 36, 48 and 72 h. Coelimycin cluster **(A)**. Desferroxamine cluster **(B)**. Coelichelin cluster **(C)**. Deoxysugar cluster **(D)**. Terpenoid biosynthesis cluster **(E)**. Others **(F)**. Protein identifiers are indicated as SCO numbers for both strains and by predicted functions. The quantification methods are displayed in the vertical bar indicating proteins quantified by Spectral Counting (orange) or XIC (black).

A total of 80 proteins belonging to 17 secondary metabolite biosynthetic pathways showed a statistically significant abundance change according to strain, medium and/or time (Figs. 9–10). Proteins belonging to the canonical NRPS cluster CDA (SCO3210-SCO3249, 10/39 proteins), the hybrid NRPS/PKS cluster RED (SCO5877-5801, 6/21 proteins) were detected at the three time points whereas those of the PKS cluster ACT (SCO5071-5092, 18/21 proteins) were detected mainly at 48 h and 72 h (Fig. 9). This proteomic profile is consistent with assays of ACT and RED antibiotics (Fig. 1D). Proteins related to γ-butyrolactone signaling, SCO6264 (FabG-like reductase), SCO6265 (ScbR γ-butyrolactone binding regulator) and SCO0608 (ScbR-like γ-butyrolactone binding regulator) that were shown to play a positive role in the regulation of CDA, RED and ACT biosynthesis^62,63^ showed a similar expression pattern as that of these antibiotics biosynthetic pathways. Proteins involved in lantibiotic (SCO6928) and dipeptide (SCO6431) biosynthesis were also far more abundant in *SC* but mainly on glycerol (Fig. 9). Proteins of the deoxy sugar cluster (SCO0381-SCO0401) were only slightly more abundant in *SC* than in *SL* but mainly on glucose (Fig. 10D).

In contrast, all proteins of the coelimycin cluster (SCO6273-SCO6288, cluster C)^64,65^ were far more abundant in *SL* on both carbon sources but mainly at 36 h (Fig. 10A). Proteins involved in terpene or hopene as well as in geosmin (SCO6073)^66^ and germicidine (SCO7221)^67^ biosynthesis were also far more abundant in *SL* at late time points and especially on glycerol (Fig. 10E,F). These molecules are synthetized from acetyl/malonylCoA and their active synthesis in *SL* is consistent with the glycolytic metabolism of this strain. Furthermore, interestingly most proteins of the deferoxamine (SCO2782-85, synthetized from lysine)^68^ and the coelichelin (SCO0489-SCO0499, synthetized from ornithine)^69^ clusters were more abundant in *SL* on both carbon sources at late time points or throughout growth (Fig. 10B). The expression of the deferoxamine (*desABCD*) cluster is known to be under the negative control of DmdR1 (SCO4394) whose repressing effect is relieved in conditions of iron deprivation^70^. The much lower expression of *desA/sco2782* as well as of *cchB/sco0498* of the coelichelin cluster in the *phoP* mutant in qRT-PCR suggested that they might be under the positive control of PhoP. However, inspection of their promoter regions did not reveal the presence of any canonical Pho-box whereas the inspection of the promoter region of DmdR1 revealed the presence of a rather convincing Pho box downstream of the transcriptional start site in position of road block (Fig. S6C6). qRT-PCR experiment confirmed the significantly higher expression of DmdR1 in the *phoP* mutant of *SL* than in the wild type strain (Fig. S6B6). The high expression of the negative regulator DmdR1 in the *phoP* mutant of *SL* (and in *SC*) is likely to be responsible for the very low expression of *desA* and *cchB* in this strain (as well as in *SC*) (Fig. S6B7–8). The negative regulation of DmdR1 expression by PhoP makes biological sense since divalent cations such as iron are often co-transported with phosphate^71,72^ and decreased Pi uptake in conditions of Pi limitation, might not provide sufficient iron to fulfill cellular needs. Adaptation to such iron deprivation would require the synthesis of high affinity siderophores under the indirect positive control of PhoR/PhoP.

## Discussion

It has long been known that Pi limitation is a major trigger of antibiotic production in *Streptomyces*^73^. Consistently the two component system PhoR/PhoP that, in condition of Pi limitation, controls positively Pi supply, has a negative impact on antibiotic production^74^ whereas its disruption has a positive impact on these productions^75–78^. The revelation by our proteomic study of the lower abundance of the sensor kinase PhoR (SCO4229) and its cognate response regulator PhoP (SCO4230) in the antibiotic producer, *SC*, compared to the none producer, *SL*, is consistent with this ancient knowledge. Indeed, the tuning down of the regulatory role of PhoR/PhoP in *SC* was shown to be correlated with low abundance of proteins involved in Pi scavenging and uptake leading to severe Pi limitation and thus energetic stress. However, paradoxically, *SC* is characterized by a high ATP content consistent with the specific up-regulation of enzymes of the TCA or of amino acids degradative pathways that generate reduced co-factors (Figs. 5 and S5). The re-oxidation of the latter by the respiratory chain likely contributes to the high ATP content of *SC*. We propose that such activation of the oxidative metabolism constitutes a homeostatic process to re-establish the energetic balance of *SC*^14,79^. In this context, available acetylCoA and nitrogen would be used to fuel the TCA, precluding TAG biosynthesis in *SC*. Free Pi is also needed to achieve oxidative phosphorylation and consistently *SC* was shown to consume its polyphosphate stores more actively than *SL*^14^. In contrast, the predicted lower iron and N availability in *SL* compared to *SC* is predicted to lead to a slowing down of the TCA and thus to a reduced consumption of acetylCoA by the latter. *SL* is thus characterized by a glycolytic metabolism allowing TAG accumulation^14^. Interestingly the *ppk* mutant of *SL* is characterized, as *SC*, by a low TAG content and strong antibiotic production^14,80^. In condition of Pi limitation, Ppk catalyzes the regeneration of ATP from ADP and polyphosphate.^80,81^. As *SC*, the *ppk* mutant suffers from energetic stress and triggers homeostatic mechanisms comparable to those found in *SC*^13^^,14^.

However, we think that the generation of ATP by the oxidative phosphorylation cannot last in *SC* since, at some point, *SC* would face a situation of severe Pi limitation, especially after exhaustion of its polyphosphate stores^14^. Interestingly, the production of the antibiotics CDA and RED occurs in this context. The production of the ionophore CDA^82^ coincides with the transition phase, a phase of growth arrest characterized by extensive breakage of cellular macromolecules^83^ whereas that of RED^84^ occurs at bit later. RED was shown to create damage to the membrane and to promote cell death and lysis of a fraction of the population^85^. Programmed Cell Death (PCD) is well documented in *Streptomyces* and both CDA, as RED, are likely to play a role in this process^79,86^. Death and lysis of a fraction of the population would provide nutriments, and especially phosphate, to the surviving population. Indeed some putative nucleases (SCO1182, SCO3151 and SCO6341) as well as the adenosine deaminase (SCO4901)^87^ that are involved in nucleic acid and nucleotide degradation were far more abundant (up to 10 fold) in *SC* than in *SL*, at all time points on both carbon sources (Fig. S3). Interestingly the genes *sco6638*, *sco5329* and *sco0324* encoding putative endonuclease or nucleosidase are present and expressed in *SC* but absent or truncated in the *SL*. The abundance of these proteins in *SC* suggests the occurrence of cell lysis and the recycling by this strain of the phosphate present in its nucleic acids. Another indication of cell lysis is the high abundance of twelve proteases early in growth on both carbon sources in *SC* (Fig. S4) as well as the 3 to 7 fold higher abundance of the 1,4-beta-N-acetylmuramidase (SCO6466) and alpha-mannosidase (SCO0948) involved in cell wall degradation in *SC* compared to *SL* at early time points (File S2). The degradation of these cellular constituents and the resulting supply of nutriments would support the temporary activation of the oxidative metabolism of this strain to restore its energetic balance^79^.

Nonetheless, at some point a situation of Pi starvation would occur and impair the proper functioning of the respiratory chain. This would result into the leakage of electrons toward secondary acceptors and thus the generation of oxidative stress (ROS/RNS). Since we previously demonstrated that the onset of ACT biosynthesis coincides with an abrupt drop in the intracellular ATP concentration in *SC*^14^, we propose that the benzochromane quinone, ACT, as other molecules possessing quinone groups (melanine, humic acid …)^88^, might act as electron acceptor, reducing electron flow through the respiratory chain and thus respiration efficiency and ATP generation^79^. Once charged with electrons, the resulting unstable semiquinone would be reduced by the NADPH-dependent quinone or flavin reductases of the SoxR regulon whose expression is induced by ACT^89–91^. In condition of phosphate limitation, ACT would thus somehow contribute to the regulation of the energetic state of the bacteria, reducing ATP generation in order to adjust it to low Pi availability^79^. Furthermore, as electron acceptor, ACT would also reduce oxidative stress and the unexpected low expression of the SigR regulon in *SC* might be due to the proposed anti-oxidant function of ACT. This hypothesis led to the prediction that a *SC* mutant strain deleted for the ACT cluster^92^ would be more sensitive to diamide stress than the original strain. This prediction was tested and confirmed (Fig. S8). Interestingly, some reports in the literature mention that oxidative stress is an important trigger of antibiotic production^93–95^ and that some antibiotics (for example chromomycin), have anti-oxidant properties^96^ and thus constitute an adaptive response to oxidative stress.

This study also revealed that some proteins belonging to carbon and iron metabolism had a very different abundance in *SL* and in its *phoP* mutant suggesting that they might constitute novel PhoP regulatory targets. It may seem surprising that these targets were not identified in previously studies^22,24^. The expression of PhoR/PhoP being low in *SC*, the difference of expression of some PhoP targets between the original strain and its *phoP* mutant might have been too small to allow the detection of all PhoP targets. The belonging of these targets to the Pho regulon has to be confirmed by site directed mutagenesis and EMSA but even in absence of such confirmation it is of interest to discuss the possible consequences of the difference of abundance of these proteins on the cellular metabolism of the strains. The reduced abundance of SCO7511 (GAPDH) and SCO4209 (PGM) in *SL* compared to *SC* is predicted to create a bottleneck leading to the accumulation of the triose phosphates DHAP and GA3P that can be easily converted into Gly3P, the necessary precursor of membrane phospholipids (PL) and TAG. Such bottleneck would not exist in *SC* and these triose phosphates will be metabolized through subsequent glycolytic steps. In consequence, upon growth on glucose, *SC* will not generate sufficient amounts of Gly3P for PL synthesis to support active growth and TAG biosynthesis will be impaired. The restoration of a growth rate comparable to that of *SL* and of the ability of *SC* to accumulate TAG in the presence of glycerol supports this hypothesis (Fig. 1A). Furthermore, the high abundance of enzymes catalyzing the two last steps of glycolysis in *SL*, the enolase SCO7638 and the pyruvate kinase SCO2014 would contribute to the generation of acetylCoA that, together with enhanced Gly3P availability mentioned above, would support TAG accumulation in *SL*. In *SC*, the lower abundance of these enzymes might lead to lower acetylCoA generation than in *SL* and thus to a reduced ability to synthesize fatty acids. In addition, the very low abundance of the Acyl Carrier Protein (ACP) SCO2389 that belongs to the FasR operon^51^ in *SC* compared to *SL* is also likely to contribute to the limited fatty acid synthesis of this strain. Overall, these differences in protein abundance may play a part in the poorly understood accumulation of TAG under conditions of limitation of Pi in *SL*^14^ as well as in other microorganisms^15,97^. At last, the putative bottleneck in the glycolytic flux generated by the low abundance of SCO7638 and SCO2014 in *SC* might contribute to the accumulation of 3-phosphoglycerate, a direct precursor of the amino acids, serine and glycine/phenylglycine used for CDA and/or RED biosynthesis.

## Conclusion

Our study revealed the nature of the metabolic features underlying the drastically different abilities of *SL* and *SC* to produce antibiotics. We believe that these differences are likely to be due, at least in part, to the weaker expression of PhoR/PhoP in *SC* compared to *SL* leading to the relief of the indirect negative impact that PhoR/PhoP exerts on antibiotic production^75,98^. However, since the causes of the low expression of PhoR/PhoP in *SC* remain to be clarified further investigation would be needed to confirm this hypothesis. Little is known on the regulation of PhoR/PhoP expression in *Streptomyces* besides that PhoP auto-controls positively the expression of the *phoR/phoP* operon from a promoter located upstream of *phoR*^43^ while the PhoU regulator plays a negative role in the regulation of this operon^99^. *phoP* is also expressed from its own promoter located at the end of *phoR*^43^ but very little is known concerning the control of *phoP* expression from this promoter. A unique study indicated that the expression of *phoP* was negatively regulated by large ATP binding regulators of the LuxR family (LAL), SC07173 and SCO0877, to a lesser extent^100^. We propose that the LAL regulators that sense ATP repress PhoP expression when the intracellular ATP concentration is high (high Pi availability) and this repression would be relieved when ATP concentration falls below a certain threshold (low Pi availability). Since the ATP content of *SC* is high in Pi limitation as in proficiency^14^, the LAL and ATP dependent repression of *phoP* expression would be maintained in this strain even in Pi limitation. Considering the low expression of most glycolytic genes tested in *SC* it is tempting to speculate that the regulation of glycolysis is altered in this strain and promotes the use of the TCA that generates abundant ATP. This high ATP content would be responsible for the low expression of PhoR/PhoP that itself would re-inforce the oxidative metabolism of this strain.

## Materials and Methods

Most of the procedures described below were set up and used in the doctoral work of Millan-Oropeza^101^.

### Bacterial growth

Spores of *S. coelicolor* M145, a derivative of the wild-type strain A3(2) lacking the plasmids SCP1 and SCP2^9^ and *S. lividans* TK24^11^ were prepared from solid SFM medium (https://actinobase.org/index.php?title=SFM). *Streptomyces* strains were grown on solid R2YE medium (https://actinobase.org/index.php?title=R2YE) devoided of sucrose, limited in phosphate (1 mM, no K_2_HPO_4_ added) and supplemented with glucose 50 mM or glycerol 100 mM as major carbon sources. 10^6^ spores were plated on the surface of cellophane disks (Focus Packaging & Design Ltd, Louth, UK) laid down on the top of agar plates and incubated at 28 °C in darkness. Cell growth was assessed by dry cell weight every 12 h until 72 h of culture. To do so, four independent biological replicates of mycelial lawns were collected with a spatula for each strain and for each culture time, washed twice with deionized water, lyophilized and weighted.

### Total proteins extraction and digestion

Proteomic analysis was carried out at three culture times: 36, 48 and 72 h. Four biological replicates were collected for each strain cultivated in the two different media and for the three culture times. The total number of samples was thus 48 (2 strains × 2 media × 3 culture times × 4 biological replicates (Fig. S9). Half of the mycelial lawns obtained from each of the four replicates was collected with a spatula, washed twice with 50 mM Tris-HCl pH = 7.8 ice cold buffer and suspended in 3 mL of lysis buffer containing 6 M urea (Sigma Aldrich, U5378), 2 M thiourea (Sigma Aldrich, T8656), 5 mM DTT (Sigma), 0.1 M TRIS-HCl pH = 8 and 150 µL of a proteases inhibitor cocktail (Sigma Aldrich, P8465). Cells were broken using a cell disruptor (Constant systems Ltd. One shot model) at 2.6 Kbars and cells debris were removed by centrifugation (15 min at 7100 g, 4 °C). Soluble protein concentrations were measured using the 2-D Quant kit protocol (GE Healthcare Life Sciences, 80-6483-56). Aliquots of 80 µg of total protein extracts were supplemented with RapiGest^TM^ (Waters, 186001860) to obtain a final concentration of 0.1% m/v. Such protein extracts were used for the proteome analysis.

The total protein extracts (80 µg) were alkylated with iodoacetamide (10 mM final concentration) in the dark for 1 h. Samples were subsequently digested in-solution for 3 h at room temperature with Lysyl-Endopeptidase (Wako, 125-05061) at a 1:100 (w/w) protein ratio. Then, following a 5-fold dilution with deionized water, they were digested with 80 μg of sequencing-grade modified trypsin (Promega) with 1:100 protein ratio at 37 °C overnight^102^. To quench the digestion, the pH of the peptide mixtures was adjusted to 2 by adding trifluoroacetic acid (TFA). The resulting peptide mixtures were pre-cleaned with a Strata-X column (Phenomenex, ref. 8B-S100-TAK). Columns were washed with 1.5 mL of washing buffer (containing 3% acetonitrile (ACN) and 0.06% glacial acetic acid). The peptide mixtures were charged into the columns, followed by three washing steps of 500 µL. Elution of peptides was achieved using 600 µL of elution buffer (40% ACN and 0.06% glacial acetic acid). The resulting samples were concentrated under vacuum to dryness and re-suspended in 320 μl of 0.1% TFA and 2% ACN for analysis in a high-resolution mass spectrometer.

### LC-MS/MS analysis

MS analyses were performed on a Dionex U3000 RSLC coupled to an Orbitrap Fusion™ Lumos™ Tribrid™ mass spectrometer (Thermo Fisher Scientific). Four μl (at 0.25 µg peptides/ml) containing 1 μg of peptides were injected and separated using a 75-μm × 500-mm Acclaim PepMap RSLC column packed with 3-μm diameter superficially porous particles (Thermo Scientific). The gradient used was 0–45% of 80% ACN/0.1% formic acid over 180 min at a flow rate of 300 nl/min. LC-MS/MS analysis was performed utilizing a nanospray ionization source and the eluted peptides were ionized by applying 2.4 kV in positive mode. MS scans were performed at 120,000 resolution, m/z range 400‒1,500. MS/MS analysis was performed in a data-dependent mode, with a top speed cycle of 3 s for the most intense double or multiple charged precursor ions. Ions in each MS scan over threshold 10,000 were selected for fragmentation (MS2) by collision-induced dissociation (CID) for identification at 35% and detection in the ion trap followed by a top speed MS2 fragment ions. Precursors were isolated in the quadrupole with a 0.7 m/z window and dynamic exclusion within 10 ppm during 25 s was used for m/z-values already selected for fragmentation. The AGC targets are 4 × 10 ^5^ and 1 × 10 ^4^ and maximum ion filling times of 50 ms and 50 ms for MS and MS/MS respectively. Polysilaxolane ions m/z 445.12002, 519.13882 and 593.15761 were used for internal calibration.

### Proteins identification

A custom FASTA format database was constructed from the genomes of *S. coelicolor* M145 and *S. lividans* TK24 downloaded from The Universal Protein Resource (http://www.uniprot.org/, June 22th 2011, 7810 and 7551 entries respectively). Unique labels were attributed to proteins encoded by orthologous genes that fulfilled an E-value <10E-10 as a top score after BLASTP^103^ for both strains. This resulted in 6911 orthologous protein pairs, and 1539 strain-specific proteins (899 specific proteins for *S. coelicolor* and 640 specific proteins for *S. lividans*). The sub-cellular localization of each protein was predicted from the LocateP database^104^. The functional categories were assigned as described in Kegg^105^, BioCyc^106^ and STREP DB databases (https://www.sanger.ac.uk/resources/downloads/bacteria/streptomyces-coelicolor.html). A database of common contaminants was also used for the analysis. Database searches were performed using the X!Tandem algorithm (version 2015.12.15; http://www.thegpm.org/TANDEM/, December 15th 2015) implemented in the open source search engine X!TandemPipeline version 3.4.2^107^. Enzymatic cleavage was declared as a trypsin digestion with one possible miss cleavage. Carboxyamidomethylation of cysteine residues and oxidation of methionine residues were set to static and possible modifications, respectively. Precursor and fragment mass tolerance was 10 ppm. Identified proteins were filtered and grouped using X!TandemPipeline. Data filtering was achieved according to a peptide E-value <0.05, protein log (E-value) < –2 and to a minimum of two identified peptides per protein. Using such filtering criteria, the peptides and proteins false discovery rates (FDR) were estimated to 0.13% and 0.66% respectively (File S1). MS data were deposited online on the public database PROTICdb^108,109^ in the following URL: http://moulon.inra.fr/protic/strepto_gly_glu.

### Peptide quantification based on extracted ion current

Peptides were quantified based on XIC using MassChroQ 2.1.4^110^. XIC extraction was performed using a peak detection threshold between 30,000–50,000 range of 10 ppm (File S3). The peptide intensities thus obtained were log10-transformed for further data analyses. Peptides showing a standard deviation of retention time higher than 20 s were removed.

Normalization was performed in order to remove systemic biases between samples as well as more complex variation resulting from transient stochastic events during LC-MS/MS run, such as ESI instability. For this, we used a local normalization method adapted from Lyutvinskiy, *et al*.^111^. Briefly, intensity deviation between a given sample and a sample chosen as reference was computed for each peptide-charge quantified in both samples. The values of intensity deviation thus obtained were ordered according to the peptides’ retention time and smoothed using the smooth.spline function in R^112^. The smoothed values of intensity deviation were used as correction factors to normalize the samples. The intensities of the peptides-charges that were present in the sample to be normalized and absent from the reference sample were corrected considering that intensity deviation was similar for temporally neighboring peptides (Fig. S10). Peptides that belonged to multiple proteins were removed. To be considered in reproducibility tests the peptides have been quantified: (1) in at least 2 replicates per strain × media × culture time combination, and (2) in 12 of the 12 possible strain × media × culture time combinations. In order to remove peptides whose intensity profiles deviated from the average profile of the peptides from the same protein, Pearson-correlation coefficients were computed for each pair of peptides belonging to a same protein. For each protein, a reference peptide was chosen as the peptide showing the highest number of significant correlations. Peptides correlated to the reference peptide with a coefficient of correlation >0.75 were kept for further analysis.

### Detection of protein abundance changes

To detect protein abundance changes, two protein quantification methods were used. As recommended by Blein-Nicolas and Zivy^113^, we first performed a rough protein quantification using Spectral Counting in order to detect semi-quantitative and presence/absence variations, then we performed a finer quantification using a XIC-based approach in order to detect smaller abundance changes.

For Spectral Counting, proteins were filtered by selecting those showing a minimal difference of 10 spectra when comparing the average of spectra for the different strain × medium × culture time combinations. For each protein, the abundance in number of spectra was modeled using the following generalized linear mixed model (GLM) with a Poisson distribution:

$$S{C}_{jklm}=\mu +{S}_{j}+{M}_{k}+{T}_{l}+{(SxM)}_{jk}+{(SxT)}_{jl}+{(MxT)}_{kl}+{(SxMxT)}_{jkl}+{R}_{m}+{\varepsilon }_{jklm}$$

$$where\,{{\epsilon }}_{jklm}N(0,{\sigma }_{\theta }^{2})$$

*SC*_*jklm*_ is the number of spectra measured in strain *j*, growth medium *k*, culture time *l* and replicate *m*. *S*_*j*_ represents the effect due to strain *j*. *M*_*k*_ represents the effect due to medium *k. T*_*l*_ represents the effect due to culture time *l*. (*SxM)*_*jk*_ represents the effect due to strain *j* × growth medium *k* interaction. (*SxT)*_*jl*_ represents the effect due to strain *j* × culture time *l* interaction. (*MxT)*_*kl*_ represents the effect due to growth medium *k* × culture time *l* interaction. (*SxMxT)*_*jkl*_ represents the effect due to strain *j* × growth medium *k* × culture time *l* interaction. *R*_*m*_ represents the effect due to biological replicate *m*. *ε*_*jklm*_ is the residual error. Protein abundance changes were detected by analysis of variance (ANOVA) using a Chi-square test.

For the XIC-based approach, the effects of the strain, growth medium and of the culture time were tested for each protein by modeling the normalized peptide intensities using a mixed-effects model derived from Blein-Nicolas and Zivy^113^.

$$\begin{array}{c}{I}_{jklm}={\rm{\mu }}+{S}_{j}+{M}_{k}+{T}_{l}+{(SxM)}_{jk}+{(SxT)}_{jl}+{(MxT)}_{kl}+{(SxMxT)}_{jkl}+{R}_{m}\\ \,\,\,\,\,\,\,\,\,\,\,+\,{\theta }_{jklm}+{\varepsilon }_{jklm}\,where\,{\theta }_{jklm}N(0,{\sigma }_{\theta }^{2})\end{array}$$

$${{\epsilon }}_{jklm}N(0,{\sigma }_{\theta }^{2})$$

*I*_*jklm*_ is the peptide intensity measured in strain *j*, growth medium *k*, culture time *l* and replicate *m*. *S*_*j*_ represents the effect due to strain *j*. *M*_*k*_ represents the effect due to medium *k. T*_*l*_ represents the effect due to culture time *l*. (*SxM)*_*jk*_ represents the effect due to strain *j* × growth medium *k* interaction. (*SxT)*_*jl*_ represents the effect due to strain *j* × culture time *l* interaction. (*MxT)*_*kl*_ represents the effect due to growth medium *k* × culture time *l* interaction. (*SxMxT)*_*jkl*_ represents the effect due to strain *j* × growth medium *k* × culture time *l* interaction. *R*_*m*_ represents the effect due to biological replicate *l*. *θ*_*jklm*_ represents the technical variation due to sample handling and injection in the mass spectrometer. *ε*_*jklm*_ is the residual error. Parameters were estimated by maximizing the restricted log-likelihood (REML method). Protein abundance changes were detected by ANOVA.

For both methods (XIC and Spectral Counts), the obtained p values were adjusted for multiple testing by the Benjamini-Hochberg approach^114^. The abundance of a given protein was considered significantly variable when the adjusted p value was <0.05.

### Data analysis

Descriptive analysis of the protein abundances was performed using Principal Component Analysis (PCA) and heatmap representations. Heatmaps were constructed using hierarchical clustering based on Euclidean distances. For proteins that were quantified with both methods (XIC and Spectral Counts), only the values obtained by XIC-based approach were used in the heatmap constructions since the latter is a finer quantitative approach compared to the Spectral Counts-based method. Among the dataset, 5 samples of SL, 2 grown on glucose (one harvested at 48 h and one at 72 h) and 3 grown on glycerol (harvested at 36, 48 and 72 h), as well as one sample of SC grown on glucose at 48 h were excluded from the analysis because they showed inconsistent LC-MS/MS results. This decision was motivated by the significantly lower number of peptides identifies in the six samples compared to most of the other samples (mainly < 40,000 peptides, Fig. S1A) as well as PCA results (Fig. S1B-E). The PCA performed, based on protein abundance estimated from peptide intensities (Fig. S1B), and spectral counts (Fig. S1C), showed that these six samples were separated from the other groups. The fact that the independent replicates of the 42 remaining samples were grouped together (Fig. S1D-E) suggested that an experimental problem had affected the protein content of the excluded samples. Temporal proteomic profiles were constructed using the relative protein abundances obtained from XIC and Spectral Counts approaches of those proteins showing significant abundance variation, these values were scaled using self-organizing tree algorithm (SOTA) clustering. Proteins quantification, statistical methods and data analysis were conducted in R 3.3.2^112^, using the following packages: ade4, clValid, ggplot2, lattice, lme4, made4, nlme and reshape2^115^.

### Esterified fatty acids quantification by GC/MS

Total lipid derived fatty acids methyl esters (FAMES) were quantified from samples of lyophilized *Streptomyces* mycelium (1 mg). The method of trans-esterification was adapted from Lepage and Roy^116^ and described in detail previously^117^. Four biological replicates were conducted for each condition.

### Assay of ACT and RED production

Extracellular ACT as well as intracellular ACT and RED were quantified from four individual plates of each strain × growth medium × culture time condition. To quantify extracellular ACT, the volume of R2YE agar medium corresponding to an individual plate was cut into small pieces using a spatula and allowed to diffuse in 5 mL water for 2 h at 4 °C. The first eluate was transferred into a new tube, and 5 mL of water was added again to the agar medium and allowed to diffuse for 2 h at 4 °C. The two eluates (10 mL) were pooled filtered through a 0.2 µm polyethersulfone membrane (Pall Life Sciences) prior to analysis. 500 µL of HCl 4 M were added to 1.5 mL of the final eluate, the remaining eluate was immediately stored at −80 °C for further analyses. The mixture was incubated on ice for 10 min to allow ACT precipitation. Precipitated ACT was collected by centrifugation (17900 g for 10 min). Supernatants were discarded and the resulting pellets were suspended in 1 mL of KOH 1 M. Optical density of the solution was determined at 640 nm in a Shimadzu UV-1800 spectrophotometer using KOH 1 M as blank^118^.

Approximately 10 mg of lyophilized mycelium were used to quantify intracellular RED and ACT by adding 1 mL of methanol or 1 mL of KOH 1 M in 2 mL tubes, respectively. Cells were disrupted using a MP Biomedicals Fast-Prep 24 System (2 cycles at 5 m/s during 30 seconds each cycle). Supernatants were collected after centrifugation (17900 g for 10 min). The methanolic extract of RED was acidified with the addition of 1 mL of HCl 1 M, and optical density was measured at 530 nm against a blank constituted of methanol: HCl 1 M (1:1 ratio in volume). Intracellular extracts of ACT were measured at a wavelength of 640 nm in a Shimadzu UV-1800 spectrophotometer using KOH 1 M as blank^118^.

### Glucose, glycerol, proline and phosphate quantification assays

Samples were obtained from the diffusion of R2YE agar medium in water as described above. Different molecules were quantified from the eluates. Enzymatic assays were used to determine glucose and glycerol concentrations (Kits GAHK-20 and MAK117, Sigma-Aldrich) and a colorimetric assay kit (MAK030, Sigma-Aldrich) was used to assay total free phosphate. A colorimetric method was used for proline quantification with isatin as derivatizing agent^119^. These assays were carried out in four independent biological replicates.

### Pho box search

The transcriptional level of expression of genes encoding proteins showing a significant differential protein abundance (at least two fold at 36 or 48 h) between *SL* and *SC* was determined in qRT-PCR in *SL* and its *phoP* mutant as well as in *SC* and Pho boxes were searched in the promoter regions of these genes. A canonical Pho box is usually considered as constituted of at least two rather conserved direct repeats of the sequence GTTCACCC with a distance of 9 bp between the two highly conserved CA bases pairs^22^ (Fig. S6).

### RNA preparation and qRT-PCR experiments

RNA was isolated from mycelia obtained from *S. lividans* TK24, wild type, its *phoP* mutant and *S. coelicolor* M145 grown for 40 h at 28 °C on the solid R2YE medium described above and containing either 1 mM (no K_2_HPO_4_ added) or 5 mM free Pi. In order to preserve RNA integrity, the mycelium was immediately freezed in liquid nitrogen in a solution containing denaturating guanidinium thiocyanate buffer RA1 (Macherey-Nagel), phenol-chloroform and ß-mercaptoethanol (a reducing agent). The cells were then lysed and homogenized in the presence of glass beads (diameter < 106 µm) using a Fast-Prep apparatus (Savant Instruments). Total RNA was purified using the Nucleospin RNA Kit (Macherey-Nagel), according to the manufacturer’s instructions. To remove residual DNA, a DNAse TURBO™ treatment (Invitrogen) was performed at 37 °C for one hour and total RNA was purified with the Nucleospin RNA Clean-Up kit (Macherey-Nagel). The RNA concentrations were quantified using the Nanodrop 2000 spectrophotometer (Thermo Scientific). The integrity of the RNAs was verified using the Agilent 2100 bioanalyzer with the eukaryote total RNA 6000 Nano assay (Agilent Technologies). 1 µg of total RNA was reverse transcribed in a 20 µL final reaction volume using the High Capacity cDNA Reverse Transcription Kit (Life Technologies) with RNase inhibitor and random primers following the manufacturer’s instructions. Quantitative PCR was performed on a QuantStudio 12 K Flex Real-Time PCR System (Life Technologies) with a SYBR green detection protocol. 3 ng of cDNA were mixed with Fast SYBR Green Master Mix and 750 nM of each primer in a final volume of 10 µL. The reaction mixture was loaded on 384 well microplates and submitted to 40 cycles of PCR (95 °C/20 sec; [95 °C/1 sec; 60 °C/20 sec] X40) followed by a fusion cycle to analyze the melting curve of the PCR products. A qPCR analysis in the absence of a reverse transcription step was performed on all RNA samples to check the absence of any DNA contamination. Primers were designed using the Primer-Blast tool from NCBI and the Primer Express 3.0 software (Life Technologies) (Supplementary Table 1). Specificity and the absence of multi-locus matching at the primer site were verified by BLAST analysis. The amplification efficiencies of primers were generated using the slopes of standard curves obtained by a ten-fold dilution series. Amplification specificity for each real-time PCR reaction was confirmed by analysis of the dissociation curves. Each sample measurement was made in duplicate and four independent RNA biological samples were prepared for each condition. Determined Ct values were then exploited for further analysis. Seven most stable reference genes were selected by GenEx software (MultiD) and the geometric mean of the five most stable genes (Glk/SCO2126, AspS/SCO3795, GyrA/SCO3873, GyrB/SCO3874, RpoB/SCO4654) was used to normalize the data (HrdB/SCO5820 and RecG/SCO5566 were excluded). The determination of the relative gene expression ratio was achieved using the ΔΔCt method using three biological replicates. The values of ΔΔCt of *SC* and of the *phoP* mutant of *SL* were normalized and standardized by log transforming, mean centering and autoscaling. All data were subjected to the Student test and the results were presented as the mean of delta-delta-Ct ± standard deviation P < 0.05 was considered as statistically significant^120^.

### Test of resistance to Diamide

Spores (10^6^) of *S. coelicolor* M145 and M1141, a derivative of M145 deleted for the ACT cluster^92^ were plated on the solid R2YE glucose medium described above. Five ul of the thiol oxidant diamine (1 M) was deposited just after spores plating as described previously^121^. Plates were incubated at 28 °C in darkness and pictures of the plates were taken after 72 h of incubation.

## Supplementary information

Supplementary File S1.

Supplementary File S2.

Supplementary File S3.

## References

[1] Procopio RE, Silva IR, Martins MK, Azevedo JL, Araujo JM (2012). Antibiotics produced by Streptomyces. Braz. J. Infect. Dis..

[2] Fair RJ, Tor Y (2014). Antibiotics and bacterial resistance in the 21st century. Perspect. Med. Chem..

[3] Chater KF (2016). Recent advances in understanding Streptomyces. F1000Res.

[4] Niu G, Chater KF, Tian Y, Zhang J, Tan H (2016). Specialised metabolites regulating antibiotic biosynthesis in Streptomyces spp. FEMS Microbiol. Rev..

[5] Genilloud O (2017). Actinomycetes: still a source of novel antibiotics. Nat. Prod. Rep..

[6] Xu, Z. Large-Scale Transposition Mutagenesis of Streptomyces coelicolor Identifies Hundreds of Genes Influencing Antibiotic Biosynthesis. Appl Environ Microbiol 83, (2017). 10.1128/AEM.02889-16 PMC5335527 28062460

[7] Xu, Z., Li, Y., Wang, Y., Deng, Z. & Tao, M. Genome-Wide Mutagenesis Links Multiple Metabolic Pathways with Actinorhodin Production in Streptomyces coelicolor. Appl Environ Microbiol 85, (2019). 10.1128/AEM.03005-18 PMC6585502 30709825

[8] van der Heul HU, Bilyk BL, McDowall KJ, Seipke RF, van Wezel GP (2018). Regulation of antibiotic production in Actinobacteria: new perspectives from the post-genomic era. Nat. Prod. Rep..

[9] Bentley SD (2002). Complete genome sequence of the model actinomycete Streptomyces coelicolor A3(2. Nature.

[10] Hopwood DA (2019). Highlights of Streptomyces genetics. Heredity (Edinb..

[11] Ruckert C (2015). Complete genome sequence of Streptomyces lividans TK24. J. Biotechnol..

[12] Olukoshi ER, Packter NM (1994). Importance of stored triacylglycerols in Streptomyces: possible carbon source for antibiotics. Microbiology.

[13] Le Marechal P (2013). Comparative proteomic analysis of Streptomyces lividans Wild-Type and ppk mutant strains reveals the importance of storage lipids for antibiotic biosynthesis. Appl. Env. Microbiol..

[14] Esnault C (2017). Strong antibiotic production is correlated with highly active oxidative metabolism in Streptomyces coelicolor M145. Sci. Rep..

[15] Wang Y (2018). Systems analysis of phosphate-limitation-induced lipid accumulation by the oleaginous yeast Rhodosporidium toruloides. Biotechnol. Biofuels.

[16] Roopnarain A, Gray VM, Sym SD (2014). Phosphorus limitation and starvation effects on cell growth and lipid accumulation in Isochrysis galbana U4 for biodiesel production. Bioresour. Technol..

[17] Millan-Oropeza A (2017). Quantitative Proteomics Analysis Confirmed Oxidative Metabolism Predominates in Streptomyces coelicolor versus Glycolytic Metabolism in Streptomyces lividans. J. Proteome Res..

[18] Santos-Beneit F (2015). The Pho regulon: a huge regulatory network in bacteria. Front. Microbiol..

[19] Smirnov A, Esnault C, Prigent M, Holland IB, Virolle MJ (2015). Phosphate Homeostasis in Conditions of Phosphate Proficiency and Limitation in the Wild Type and the phoP Mutant of Streptomyces lividans. PLoS One.

[20] Rodriguez-Garcia A, Barreiro C, Santos-Beneit F, Sola-Landa A, Martin JF (2007). Genome-wide transcriptomic and proteomic analysis of the primary response to phosphate limitation in Streptomyces coelicolor M145 and in a DeltaphoP mutant. Proteomics.

[21] Rodriguez-Garcia A, Sola-Landa A, Apel K, Santos-Beneit F, Martin JF (2009). Phosphate control over nitrogen metabolism in Streptomyces coelicolor: direct and indirect negative control of glnR, glnA, glnII and amtB expression by the response regulator PhoP. Nucleic Acids Res..

[22] Allenby NE, Laing E, Bucca G, Kierzek AM, Smith CP (2012). Diverse control of metabolism and other cellular processes in Streptomyces coelicolor by the PhoP transcription factor: genome-wide identification of *in vivo* targets. Nucleic Acids Res..

[23] Martin JF, Rodriguez-Garcia A, Liras P (2017). The master regulator PhoP coordinates phosphate and nitrogen metabolism, respiration, cell differentiation and antibiotic biosynthesis: comparison in Streptomyces coelicolor and Streptomyces avermitilis. J. Antibiot. (Tokyo.

[24] Sola-Landa A, Rodriguez-Garcia A, Apel AK, Martin JF (2008). Target genes and structure of the direct repeats in the DNA-binding sequences of the response regulator PhoP in Streptomyces coelicolor. Nucleic Acids Res..

[25] Sandoval-Calderon M (2015). Plasticity of Streptomyces coelicolor Membrane Composition Under Different Growth Conditions and During Development. Front. Microbiol..

[26] Wray LV, Atkinson MR, Fisher SH (1991). Identification and cloning of the glnR locus, which is required for transcription of the glnA gene in Streptomyces coelicolor A3(2. J. Bacteriol..

[27] Fink D, Weissschuh N, Reuther J, Wohlleben W, Engels A (2002). Two transcriptional regulators GlnR and GlnRII are involved in regulation of nitrogen metabolism in Streptomyces coelicolor A3(2. Mol. Microbiol..

[28] Reuther J, Wohlleben W (2007). Nitrogen metabolism in Streptomyces coelicolor: transcriptional and post-translational regulation. J. Mol. Microbiol. Biotechnol..

[29] Wang Y, Cen XF, Zhao GP, Wang J (2012). Characterization of a new GlnR binding box in the promoter of amtB in Streptomyces coelicolor inferred a PhoP/GlnR competitive binding mechanism for transcriptional regulation of amtB. J. Bacteriol..

[30] Amin R (2016). Post-translational Serine/Threonine Phosphorylation and Lysine Acetylation: A Novel Regulatory Aspect of the Global Nitrogen Response Regulator GlnR in S. coelicolor M145. Front. Mol. Biosci..

[31] He JM (2016). Direct Involvement of the Master Nitrogen Metabolism Regulator GlnR in Antibiotic Biosynthesis in Streptomyces. J. Biol. Chem..

[32] Xu Y, You D, Ye BC (2017). Nitrogen regulator GlnR directly controls transcription of genes encoding lysine deacetylases in Actinobacteria. Microbiology.

[33] Crack JC (2015). NsrR from Streptomyces coelicolor is a nitric oxide-sensing [4Fe-4S] cluster protein with a specialized regulatory function. J. Biol. Chem..

[34] Urem, M. OsdR of Streptomyces coelicolor and the Dormancy Regulator DevR of Mycobacterium tuberculosis Control Overlapping Regulons. mSystems 1, (2016). 10.1128/mSystems.00014-16 PMC5069765 27822533

[35] Falke D, Fischer M, Sawers RG (2016). Phosphate and oxygen limitation induce respiratory nitrate reductase 3 synthesis in stationary-phase mycelium of Streptomyces coelicolor A3(2. Microbiology.

[36] Bush MJ (2018). The actinobacterial WhiB-like (Wbl) family of transcription factors. Mol. Microbiol..

[37] Voskuil MI (2003). Inhibition of respiration by nitric oxide induces a Mycobacterium tuberculosis dormancy program. J. Exp. Med..

[38] Valledor L, Furuhashi T, Recuenco-Munoz L, Wienkoop S, Weckwerth W (2014). System-level network analysis of nitrogen starvation and recovery in Chlamydomonas reinhardtii reveals potential new targets for increased lipid accumulation. Biotechnol. Biofuels.

[39] Goncalves EC (2016). Nitrogen starvation-induced accumulation of triacylglycerol in the green algae: evidence for a role for ROC40, a transcription factor involved in circadian rhythm. Plant. J..

[40] Morin N (2011). Transcriptomic analyses during the transition from biomass production to lipid accumulation in the oleaginous yeast Yarrowia lipolytica. PLoS One.

[41] Smith CP, Chater KF (1988). Structure and regulation of controlling sequences for the Streptomyces coelicolor glycerol operon. J. Mol. Biol..

[42] Schniete, J. K. Expanding Primary Metabolism Helps Generate the Metabolic Robustness To Facilitate Antibiotic Biosynthesis in Streptomyces. mBio 9, (2018). 10.1128/mBio.02283-17 PMC5801464 29437921

[43] Ghorbel S, Kormanec J, Artus A, Virolle MJ (2006). Transcriptional studies and regulatory interactions between the phoR-phoP operon and the phoU, mtpA, and ppk genes of Streptomyces lividans TK24. J. Bacteriol..

[44] Gygi SP, Rochon Y, Franza BR, Aebersold R (1999). Correlation between protein and mRNA abundance in yeast. Mol. Cell Biol..

[45] Jeong Y (2016). The dynamic transcriptional and translational landscape of the model antibiotic producer Streptomyces coelicolor A3(2. Nat. Commun..

[46] Sola-Landa A, Rodriguez-Garcia A, Franco-Dominguez E, Martin JF (2005). Binding of PhoP to promoters of phosphate-regulated genes in Streptomyces coelicolor: identification of PHO boxes. Mol. Microbiol..

[47] Rodriguez E, Navone L, Casati P, Gramajo H (2012). Impact of malic enzymes on antibiotic and triacylglycerol production in Streptomyces coelicolor. Appl. Environ. Microbiol..

[48] Zhang Y, Adams IP, Ratledge C (2007). Malic enzyme: the controlling activity for lipid production? Overexpression of malic enzyme in Mucor circinelloides leads to a 2.5-fold increase in lipid accumulation. Microbiology.

[49] Ratledge C (2014). The role of malic enzyme as the provider of NADPH in oleaginous microorganisms: a reappraisal and unsolved problems. Biotechnol. Lett..

[50] Brekasis, D. & Paget M. S. B. A novel sensor of NADH/NAD+ redox poise in Streptomyces coelicolor A3(2). EMBO J. 22:4856-4865 (2003) 10.1093/emboj/cdg453 PMC212721 12970197

[51] Arabolaza A, D’Angelo M, Comba S, Gramajo H (2010). FasR, a novel class of transcriptional regulator, governs the activation of fatty acid biosynthesis genes in Streptomyces coelicolor. Mol. Microbiol..

[52] Lee, C. & Park, C. Bacterial Responses to Glyoxal and Methylglyoxal: Reactive Electrophilic Species. Int J Mol Sci 18, (2017). 10.3390/ijms18010169 PMC5297802 28106725

[53] Booth, I. R. Glycerol and Methylglyoxal Metabolism. EcoSal Plus 1, (2005). 10.1128/ecosalplus.3.4.3 26443506

[54] Newton GL, Buchmeier N, Fahey RC (2008). Biosynthesis and functions of mycothiol, the unique protective thiol of Actinobacteria. Microbiol. Mol. Biol. Rev..

[55] Daigle F (2015). A terD domain-encoding gene (SCO2368) is involved in calcium homeostasis and participates in calcium regulation of a DosR-like regulon in Streptomyces coelicolor. J. Bacteriol..

[56] Kvint K, Nachin L, Diez A, Nystrom T (2003). The bacterial universal stress protein: function and regulation. Curr. Opin. Microbiol..

[57] Chiancone E, Ceci P (2010). The multifaceted capacity of Dps proteins to combat bacterial stress conditions: Detoxification of iron and hydrogen peroxide and DNA binding. Biochim. Biophys. Acta.

[58] Facey PD (2011). The dpsA gene of Streptomyces coelicolor: induction of expression from a single promoter in response to environmental stress or during development. PLoS One.

[59] Pane-Farre J, Quin MB, Lewis RJ, Marles-Wright J (2017). Structure and Function of the Stressosome Signalling Hub. Subcell. Biochem..

[60] Kallifidas D, Thomas D, Doughty P, Paget MS (2010). The sigmaR regulon of Streptomyces coelicolor A32 reveals a key role in protein quality control during disulphide stress. Microbiology.

[61] Lee S, Kim SM, Lee RT (2013). Thioredoxin and thioredoxin target proteins: from molecular mechanisms to functional significance. Antioxid. Redox Signal..

[62] Li X (2015). ScbR- and ScbR2-mediated signal transduction networks coordinate complex physiological responses in Streptomyces coelicolor. Sci. Rep..

[63] Mehra S, Charaniya S, Takano E, Hu WS (2008). A bistable gene switch for antibiotic biosynthesis: the butyrolactone regulon in Streptomyces coelicolor. PLoS One.

[64] Bednarz B, Kotowska M, Pawlik KJ (2019). Multi-level regulation of coelimycin synthesis in Streptomyces coelicolor A3(2. Appl. Microbiol. Biotechnol..

[65] Gomez-Escribano JP (2012). Structure and biosynthesis of the unusual polyketide alkaloid coelimycin P1, a metabolic product of the cpk gene cluster of Streptomyces coelicolor M145. Chem. Sci..

[66] Jiang J, He X, Cane DE (2007). Biosynthesis of the earthy odorant geosmin by a bifunctional Streptomyces coelicolor enzyme. Nat. Chem. Biol..

[67] Chemler JA (2012). Biochemical and structural characterization of germicidin synthase: analysis of a type III polyketide synthase that employs acyl-ACP as a starter unit donor. J. Am. Chem. Soc..

[68] Barona-Gomez F, Wong U, Giannakopulos AE, Derrick PJ, Challis GL (2004). Identification of a cluster of genes that directs desferrioxamine biosynthesis in Streptomyces coelicolor M145. J. Am. Chem. Soc..

[69] Barona-Gomez F (2006). Multiple biosynthetic and uptake systems mediate siderophore-dependent iron acquisition in Streptomyces coelicolor A3(2) and Streptomyces ambofaciens ATCC 23877. Microbiology.

[70] Tunca S, Barreiro C, Sola-Landa A, Coque JJ, Martin JF (2007). Transcriptional regulation of the desferrioxamine gene cluster of Streptomyces coelicolor is mediated by binding of DmdR1 to an iron box in the promoter of the desA gene. FEBS J..

[71] Veen HW, Abee T, Kortstee GJ, Konings WN, Zehnder AJ (1994). Translocation of metal phosphate via the phosphate inorganic transport system of Escherichia coli. Biochemistry.

[72] Beard SJ (2000). Evidence for the transport of zinc(II) ions via the pit inorganic phosphate transport system in Escherichia coli. FEMS Microbiol. Lett..

[73] Martin JF, Demain AL (1980). Control of antibiotic biosynthesis. Microbiol. Rev..

[74] Martin JF (2004). Phosphate control of the biosynthesis of antibiotics and other secondary metabolites is mediated by the PhoR-PhoP system: an unfinished story. J. Bacteriol..

[75] Barreiro C, Martinez-Castro M (2019). Regulation of the phosphate metabolism in Streptomyces genus: impact on the secondary metabolites. Appl. Microbiol. Biotechnol..

[76] Barreales EG, Payero TD, Pedro A, Aparicio JF (2018). Phosphate effect on filipin production and morphological differentiation in Streptomyces filipinensis and the role of the PhoP transcription factor. PLoS One.

[77] Yang R (2015). The PhoP transcription factor negatively regulates avermectin biosynthesis in Streptomyces avermitilis. Appl. Microbiol. Biotechnol..

[78] Sola-Landa A, Moura RS, Martin JF (2003). The two-component PhoR-PhoP system controls both primary metabolism and secondary metabolite biosynthesis in Streptomyces lividans. Proc. Natl Acad. Sci. USA.

[79] Virolle M-J (2020). A Challenging View: Antibiotics Play a Role in the Regulation of the Energetic Metabolism of the Producing Bacteria. Antibiotics.

[80] Chouayekh H, Virolle MJ (2002). The polyphosphate kinase plays a negative role in the control of antibiotic production in Streptomyces lividans. Mol. Microbiol..

[81] Ghorbel S (2006). Regulation of ppk expression and *in vivo* function of Ppk in Streptomyces lividans TK24. J. Bacteriol..

[82] Lakey JH, Lea EJ, Rudd BA, Wright HM, Hopwood DA (1983). A new channel-forming antibiotic from Streptomyces coelicolor A3(2) which requires calcium for its activity. J. Gen. Microbiol..

[83] Novotna J (2003). Proteomic studies of diauxic lag in the differentiating prokaryote Streptomyces coelicolor reveal a regulatory network of stress-induced proteins and central metabolic enzymes. Mol. Microbiol..

[84] Takano E (1992). Transcriptional regulation of the redD transcriptional activator gene accounts for growth-phase-dependent production of the antibiotic undecylprodigiosin in Streptomyces coelicolor A3(2. Mol. Microbiol..

[85] Tenconi E, Traxler MF, Hoebreck C, Wezel GP, Rigali S (2018). Production of Prodiginines Is Part of a Programmed Cell Death Process in Streptomyces coelicolor. Front. Microbiol..

[86] Rioseras B, Lopez-Garcia MT, Yague P, Sanchez J, Manteca A (2014). Mycelium differentiation and development of Streptomyces coelicolor in lab-scale bioreactors: programmed cell death, differentiation, and lysis are closely linked to undecylprodigiosin and actinorhodin production. Bioresour. Technol..

[87] Pornbanlualap S, Chalopagorn P (2011). Adenosine deaminase from Streptomyces coelicolor: recombinant expression, purification and characterization. Protein Expr. Purif..

[88] Lu JM, Rosokha SV, Neretin IS, Kochi JK (2006). Quinones as electron acceptors. X-ray structures, spectral (EPR, UV-vis) characteristics and electron-transfer reactivities of their reduced anion radicals as separated vs contact ion pairs. J. Am. Chem. Soc..

[89] Dela Cruz R (2010). Expression of the Streptomyces coelicolor SoxR regulon is intimately linked with actinorhodin production. J. Bacteriol..

[90] Shin JH, Singh AK, Cheon DJ, Roe JH (2011). Activation of the SoxR regulon in Streptomyces coelicolor by the extracellular form of the pigmented antibiotic actinorhodin. J. Bacteriol..

[91] Naseer N, Shapiro JA, Chander M (2014). RNA-Seq analysis reveals a six-gene SoxR regulon in Streptomyces coelicolor. PLoS One.

[92] Gomez-Escribano JP, Bibb MJ (2011). Engineering Streptomyces coelicolor for heterologous expression of secondary metabolite gene clusters. Microb. Biotechnol..

[93] Beites T (2011). Crosstalk between ROS homeostasis and secondary metabolism in S. natalensis ATCC 27448: modulation of pimaricin production by intracellular ROS. PLoS One.

[94] Beites T (2015). Streptomyces natalensis programmed cell death and morphological differentiation are dependent on oxidative stress. Sci. Rep..

[95] Miranda RU (2014). Reactive oxygen species regulate lovastatin biosynthesis in Aspergillus terreus during submerged and solid-state fermentations. Fungal Biol..

[96] Prajapati, D., Kumari, N., Dave, K., Chatupale, V. & Pohnerkar, J. Chromomycin, an antibiotic produced by Streptomyces flaviscleroticus might play a role in the resistance to oxidative stress and is essential for viability in stationary phase. Environ Microbiol (2018). 10.1111/1462-2920.14515 30585380

[97] Wu S, Hu C, Jin G, Zhao X, Zhao ZK (2010). Phosphate-limitation mediated lipid production by Rhodosporidium toruloides. Bioresour. Technol..

[98] Romero-Rodriguez A (2018). Interplay between carbon, nitrogen and phosphate utilization in the control of secondary metabolite production in Streptomyces. Antonie Van. Leeuwenhoek.

[99] Martin-Martin, S. Self-control of the PHO regulon: the PhoP-dependent protein PhoU controls negatively expression of genes of PHO regulon in Streptomyces coelicolor. J Antibiot (Tokyo (2017). 10.1038/ja.2017.130 29089595

[100] Guerra SM (2012). LAL regulators SCO0877 and SCO7173 as pleiotropic modulators of phosphate starvation response and actinorhodin biosynthesis in Streptomyces coelicolor. PLoS One.

[101] Millan Oropeza, A. Comparative study of the proteome of* S. coelicolor* M145 and *S. lividans* TK24, two phylogenetically closely related strains with very different abilities to accumulate TAG and produce antibiotics. Université Paris-Saclay. NNT : 2017SACLS160 (2017).

[102] Wisniewski JR, Mann M (2012). Consecutive proteolytic digestion in an enzyme reactor increases depth of proteomic and phosphoproteomic analysis. Anal. Chem..

[103] Camacho C (2009). BLAST+: architecture and applications. BMC Bioinforma..

[104] Zhou M, Boekhorst J, Francke C, Siezen RJ (2008). LocateP: genome-scale subcellular-location predictor for bacterial proteins. BMC Bioinforma..

[105] Kanehisa M, Goto S (2000). KEGG: kyoto encyclopedia of genes and genomes. Nucleic Acids Res..

[106] Karp PD (2005). Expansion of the BioCyc collection of pathway/genome databases to 160 genomes. Nucleic Acids Res..

[107] Langella O (2017). X!TandemPipeline: A Tool to Manage Sequence Redundancy for Protein Inference and Phosphosite Identification. J. Proteome Res..

[108] Ferry-Dumazet H (2005). PROTICdb: a web-based application to store, track, query, and compare plant proteome data. Proteomics.

[109] Langella O (2013). Management and dissemination of MS proteomic data with PROTICdb: example of a quantitative comparison between methods of protein extraction. Proteomics.

[110] Valot B, Langella O, Nano E, Zivy M (2011). MassChroQ: a versatile tool for mass spectrometry quantification. Proteomics.

[111] Lyutvinskiy Y, Yang H, Rutishauser D, Zubarev RA (2013). In silico instrumental response correction improves precision of label-free proteomics and accuracy of proteomics-based predictive models. Mol. Cell Proteom..

[112] R Core Team. R: A language and environment for statistical computing. R Foundation for Statistical Computing, Vienna, Austria. URL https://www.R-project.org/. (2017)

[113] Blein-Nicolas M, Zivy M (2016). Thousand and one ways to quantify and compare protein abundances in label-free bottom-up proteomics. Biochim. Biophys. Acta.

[114] Benjamini Y, Hochberg Y (1995). Controlling the False Discovery Rate: A Practical and Powerful Approach to Multiple Testing. Journal of the Royal Statistical Society. Ser. B (Methodol..

[115] Culhane AC, Thioulouse J, Perriere G, Higgins DG (2005). MADE4: an R package for multivariate analysis of gene expression data. Bioinformatics.

[116] Lepage G, Roy CC (1986). Direct transesterification of all classes of lipids in a one-step reaction. J. Lipid Res..

[117] Millan-Oropeza A (2017). Attenuated Total Reflection Fourier Transform Infrared (ATR FT-IR) for Rapid Determination of Microbial Cell Lipid Content: Correlation with Gas Chromatography-Mass Spectrometry (GC-MS. Appl. Spectrosc..

[118] Kieser, T., Bibb, M. J, Buttner, M. J., Chater, K. F., Hopwood, D. A. Practical *Streptomyces* Genetics. John Innes Foundation: Norwich (2000).

[119] Long CM, Virolle MJ, Chang SY, Chang S, Bibb M (1987). J. alpha-Amylase gene of Streptomyces limosus: nucleotide sequence, expression motifs, and amino acid sequence homology to mammalian and invertebrate alpha-amylases. J. Bacteriol..

[120] Willems E, Leyns L, Vandesompele J (2008). Standardization of real-time PCR gene expression data from independent biological replicates. Anal. Biochem..

[121] Paget MS, Kang JG, Roe JH, Buttner. MJ (1998). sigmaR, an RNA polymerase sigma factor that modulates expression of the thioredoxin system in response to oxidative stress in Streptomyces coelicolor A3(2. EMBO J..

