# Supplementary material for: Expression of genes of the Pho regulon is altered in Streptomyces coelicolor
Source: Sci Rep. 2020 May 22;10:8492. doi: 10.1038/s41598-020-65087-w (PMC7244524; doi:10.1038/s41598-020-65087-w)
Supplement: Supplementary file 3 — Supplementary File S3. [file 41598_2020_65087_MOESM3_ESM.pdf]

Expression of genes of the Pho regulon is altered in *Streptomyces coelicolor*.

Aaron Millan-Oropeza [1, 2¶], Céline Henry [2¶], Clara Lejeune [1], Michelle David [1] and Marie-Joelle Virolle[1¶*]

[1] Université Paris-Saclay, CEA, CNRS, Institute for Integrative Biology of the Cell (I2BC), 91198, Gif-sur-Yvette, France.

[2] PAPPSO, Micalis Institute, INRAE, AgroParisTech, Université Paris-Saclay, Jouy-en-Josas, France.

[¶] Contributed equally

* Corresponding author. Email address: marie-joelle.virolle@i2bc.paris-saclay.fr

File S3 : MASSCHROQ quantification parameters


```
<alignments>
    <alignment_methods>
      <alignment_method id="ms2_1">
        <ms2><!--<ms2 write_time_values_output_dir="alignment_dir">-->
          <ms2_tendency_halfwindow>10</ms2_tendency_halfwindow>
          <ms2_smoothing_halfwindow>15</ms2_smoothing_halfwindow>
          <ms1_smoothing_halfwindow>0</ms1_smoothing_halfwindow>
        </ms2>
      </alignment_method>
    </alignment_methods>
    <align group_id="G1" method_id="ms2_1" reference_data_id="samp0"/>
  </alignments>
  <quantification_methods>
    <quantification_method id="quant1">
      <xic_extraction xic_type="max"><!--max : XIC on BasePeak; sum : XIC on TIC-->
        <ppm_range min="10" max="10"/><!--For XIC extraction on Da use: mz_range-->
      </xic_extraction>
      <xic_filters>
        <anti_spike half="2"/>
        <background half_mediane="5" half_min_max="20"/>
      </xic_filters>
      <peak_detection>
        <detection_zivy>
          <mean_filter_half_edge>1</mean_filter_half_edge>
          <minmax_half_edge>3</minmax_half_edge>
          <maxmin_half_edge>2</maxmin_half_edge>
          <detection_threshold_on_max>50000</detection_threshold_on_max>
          <detection_threshold_on_min>30000</detection_threshold_on_min>
        </detection_zivy>
      </peak_detection>
    </quantification_method>
  </quantification_methods>
  <quantification>
    <quantification_results>
      <quantification_result output_file="XIC_result" format="tsv"/>
      <quantification_result output_file="result.xml" format="masschroqml"
xic_traces="false"/>
    </quantification_results>
    <quantify id="q1" withingroup="G1" quantification_method_id="quant1">
      <peptides_in_peptide_list mode="post_matching"/><!--prefer 'real_or_mean' mode on
low resolution-->
    </quantify>
  </quantification>
</masschroq>
```

Table S1: List of primer used for qRT-PCR experiments

Reference gene	Gene name		Primer sequences
SCO2126	Glk	F	CAGCGCTCCACGGTCTACTT
		R	GTGATGCAGATGACGTTGC
SCO3795	AspS	F	CTGCTGATGATCTCGGGCTT
		R	CGAGCTGGTAGAACTCGCC
SCO3873	GyrA	F	GGCGACTCCTCCATCTACGA
		R	GACCATCTCCATCGACAGC
SCO3874	GyrB	F	TCGAGACCACCGACTACTCCTT
		R	TCTTGACCTCGTGCTTCTCG
SCO4654	RpoB	F	CTTCGAGCCTCCCAAGAACA
		R	TTGGTCATGAGCGGGAAGT
SCO5566	RecG (excluded)	F	TCTTCAACCGTCGACTGCAA
		R	TTCCAGGACTCCAGCTTCG
SCO5820	HrdB (excluded)	F	CTCAAGCAGATCGGCAAGGT
		R	TCCAGCAGGTGGTTCTTGG

Target gene	Gene name		Primer sequences
SCO2198	Glutamine synthetase I, GlnA	F	GTGAAGAACGTGGCCTGGAA
		R	CGTAGCCCTGCTCGTCGTAG
SCO2210	Glutamine synthetase	F	GCTCCGTTCCAAGACGAAGA
		R	GAGAAGACCGGCTTGAGCAC
SCO4139	PstB	F	CTGAAGGAGCGCTTCACGAT
		R	CCAGGTTGAAGAACGCCGTA
SCO4141	PstC	F	CCAGAACATCGCCAGCAAGT
		R	CAGCAGGGTGATGACGAACA
SCO4142	PstS	F	AGCAACATCAAGTGCGACGA
		R	GGAACCGGTCGGGTTGTAGT
SCO4144	mutT1	F	GTACGCGTCCCGTCTGGAG
		R	GCCCACCGTGAGGTTGC
SCO4145	ppk	F	AGTCCGCCAACATCAAGTGG
		R	GCTTGCAGTGGGTCTTCAGG
SCO4159	GlnR	F	ACGTACTCCGCGAAGCTGAA
		R	GTGCCGCCGAAGTAGTCGTA
SCO4228	PhoU	F	ACGCGACCATCCTGGAGAT
		R	GGTCGACGTCCTTGGTGATG
SCO4229	PhoP	F	GACGGAGAGATACGGCAGGTC
		R	CTCACGTTGGCGACGAAGTC
SCO4230	PhoR	F	GTGCTCCTCGACCTGATGCT
		R	GTCGATCTCGCTGTCCTTGG

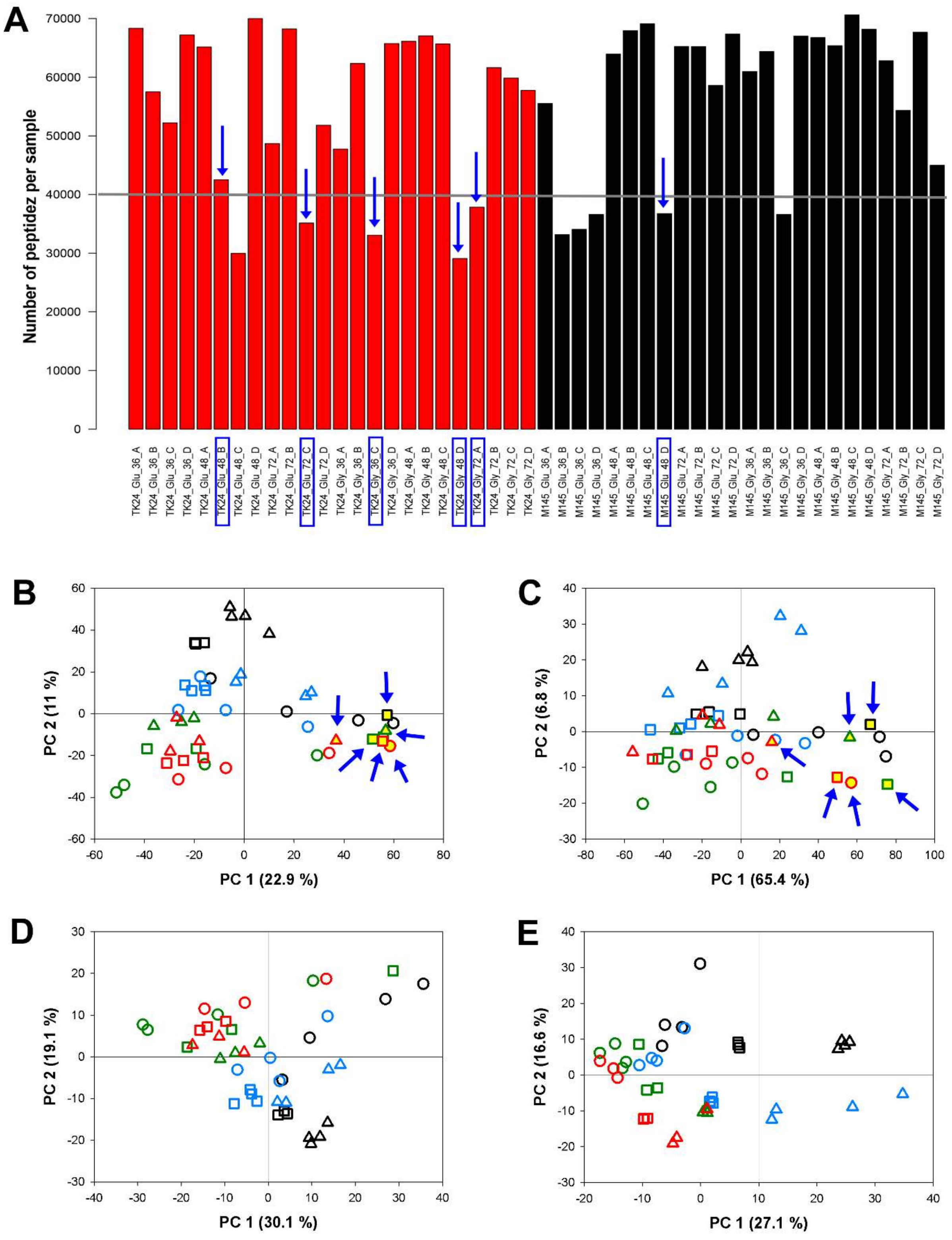

Figure S1: LC-MS/MS samples of the proteomic dataset. Number of peptides per sample (**A**) in *S. lividans* (red) and *S. coelicolor* (blue). Principal Component Analysis (PCA) of proteins abundances from peptide intensities (**B** and **D**) and spectral counts (**C** and **E**) of the proteomic dataset. PCA including dubious LC-MS/MS (**B** and **C**). PCA of the remaining 42 samples without dubious LC-MS/MS runs (**C** and **D**). Samples originated from glucose or glycerol grown cultures are indicated in black and blue for *S. coelicolor* and in green and red for *S. lividans*, respectively. Time points are represented by circles (36h), squares (48h) and triangles (72h). Arrows indicate dubious LC-MS/MS runs.

A

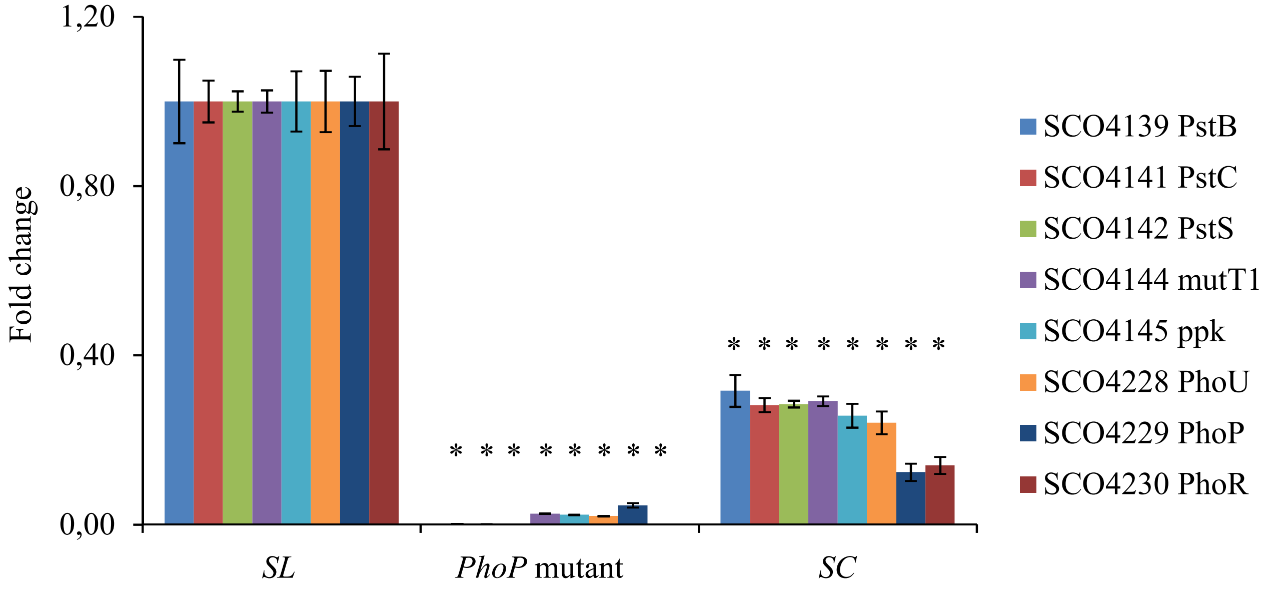

B

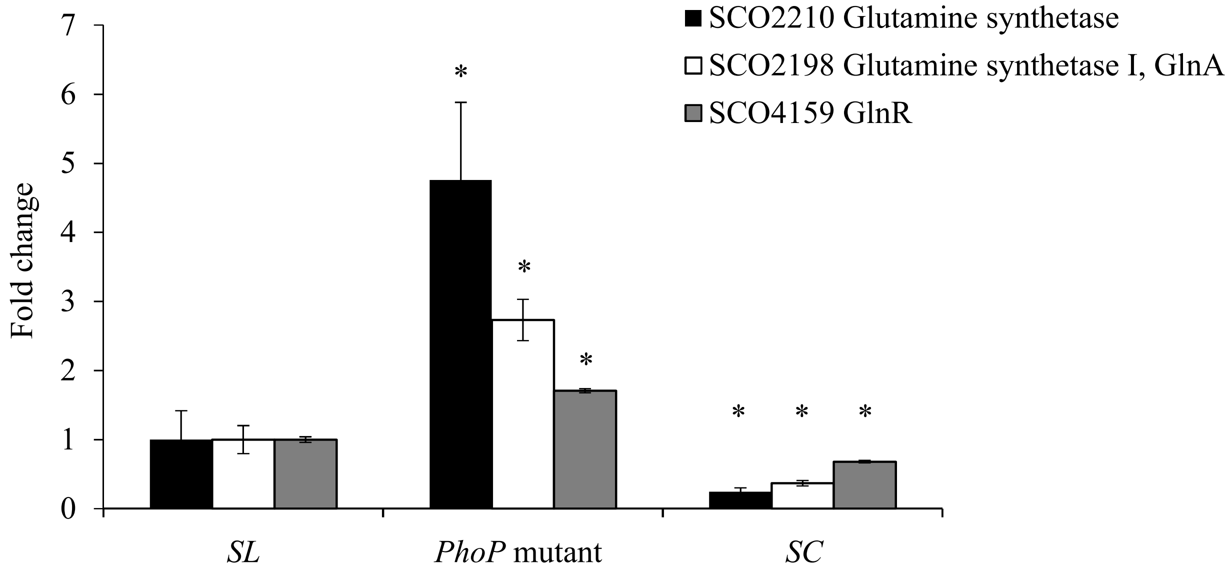

Figure S2: Determination in qRT-PCR of relative expression of genes involved in phosphate metabolism (mean with error bars representing 95% confidence interval) and known to be positively (**A**) or negatively regulated (**B**) by PhoP in the *phoP* mutant of S. *lividans* and in S. *coelicolor*. Asterisks signal significant abundance change (Student test, adjusted p value < 0.05) in comparison with SL taken as reference equal to 1. The strains were grown for 40h at 28°C on solid R2YE, limited in phosphate (1mM) and with glucose (50mM) as main carbon source.

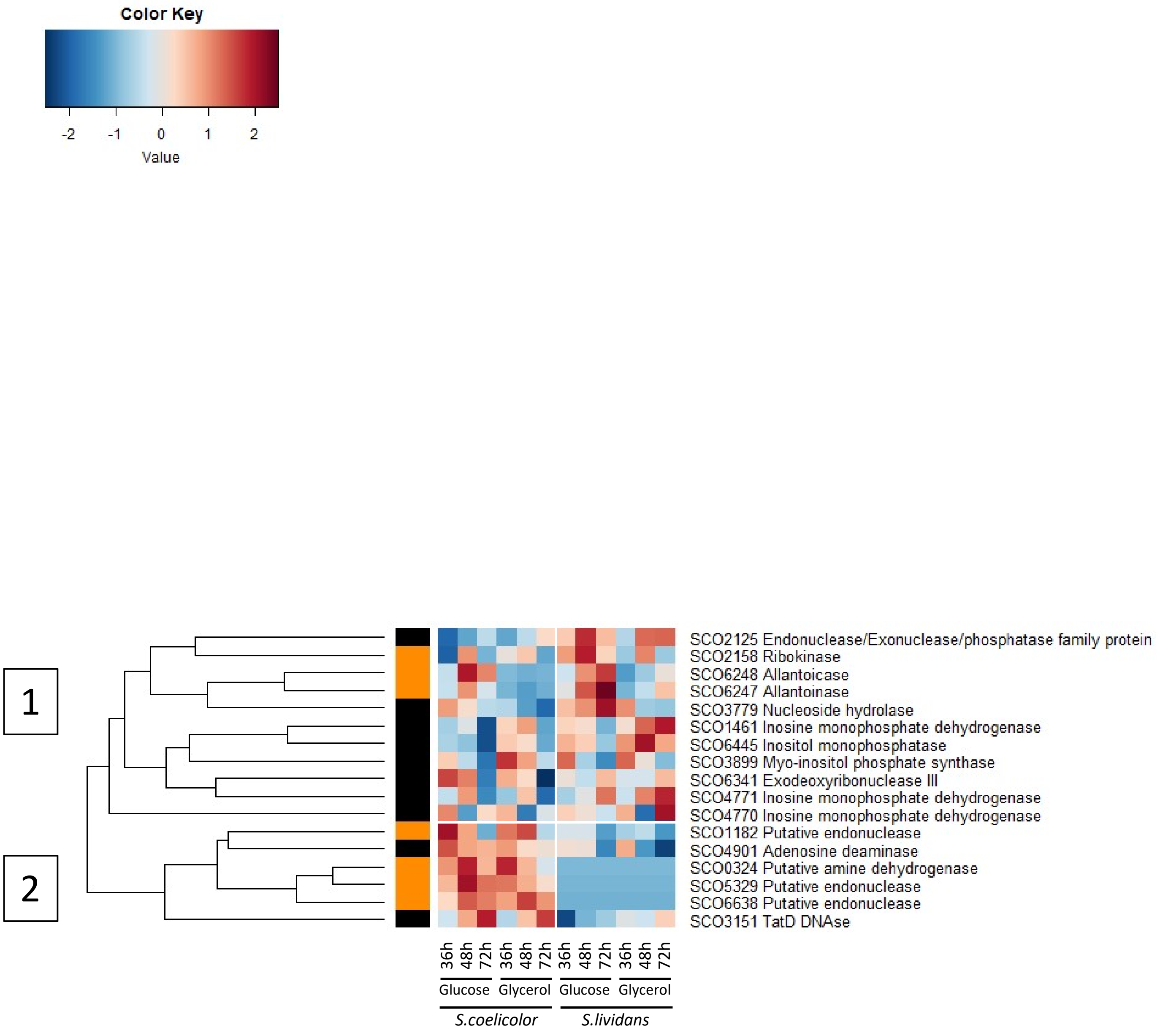

Figure S3: Heatmap representation of enzymes belonging to nucleotides degradative pathways (clusters 1 and 2) with significant abundance change (ANOVA, adjusted p value < 0.01) in *S. coelicolor* and *S. lividans* grown on R2YE medium limited in phosphate (1mM) with either glucose or glycerol as main carbon source, for 36, 48 and 72 h at 28°C. Protein identifiers are indicated as SCO numbers for both strains and by predicted functions. The quantification methods are displayed in the vertical bar indicating proteins quantified by SC (orange) or by XIC (black).

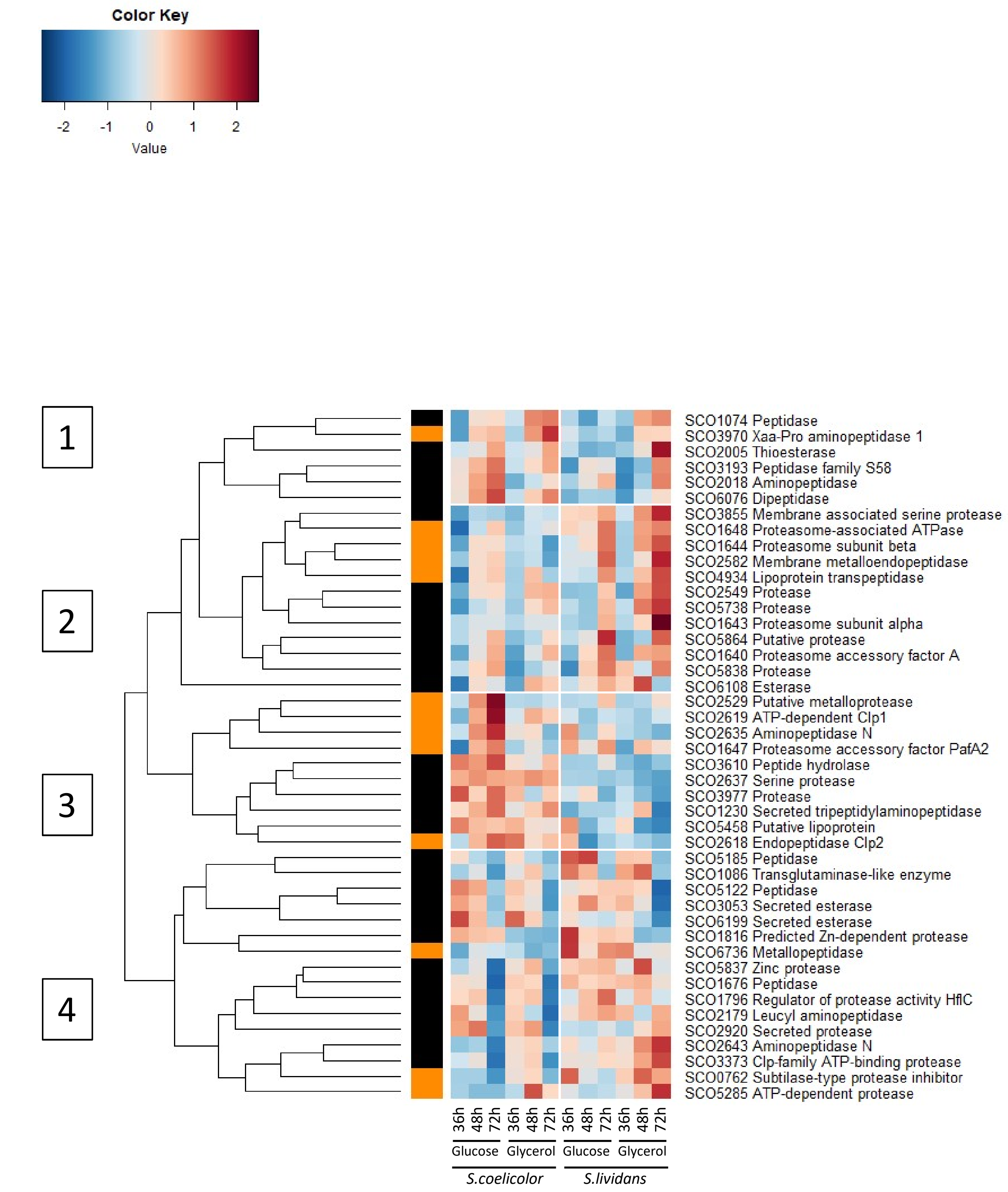

Figure S4: Heatmap representation of enzymes belonging to proteins degradative pathways (clusters 1 to 4) with significant abundance change (ANOVA, adjusted p value < 0.01) in *S. coelicolor* and *S. lividans* grown on R2YE medium limited in phosphate (1mM) with either glucose or glycerol as main carbon source, for 36, 48 and 72 h at 28°C. Protein identifiers are indicated as SCO numbers for both strains and by predicted functions. The quantification methods are displayed in the vertical bar indicating proteins quantified by SC (orange) or by XIC (black).

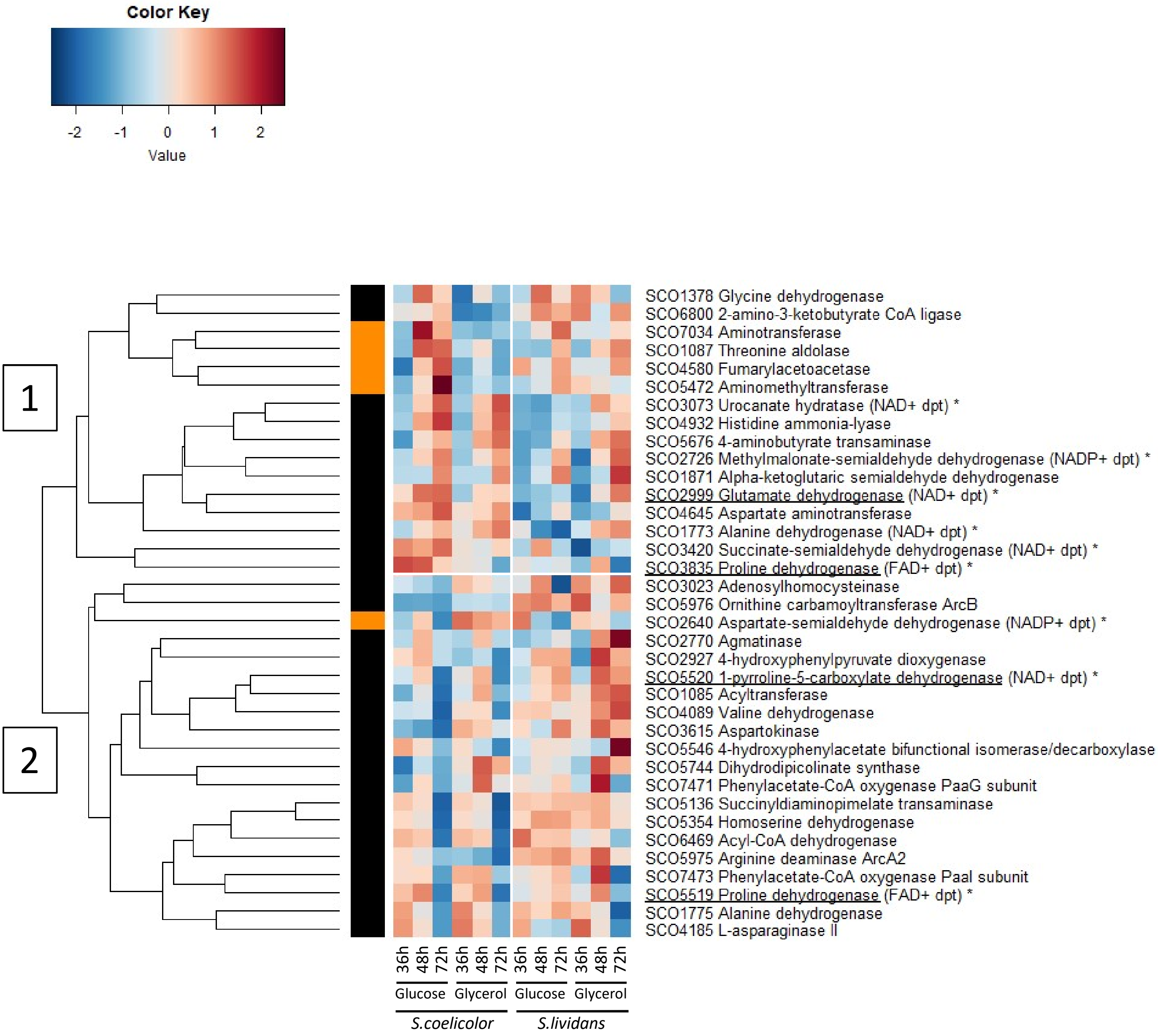

Figure S5: Heatmap representation of enzymes belonging to amino acids degradative pathways (clusters 1 and 2) with significant abundance change (ANOVA, adjusted p value < 0.01) in *S. coelicolor* and *S. lividans* grown on R2YE medium limited in phosphate (1mM) with either glucose or glycerol as main carbon source, for 36, 48 and 72 h at 28°C. Protein identifiers are indicated as SCO numbers for both strains and by predicted functions. Asterisks enzymes catalyzing a reaction generating a reduced co-factor. Proteins involved in proline degradation are underlined. The quantification methods are displayed in the vertical bar indicating proteins quantified by SC (orange) or by XIC (black).

Figure S6

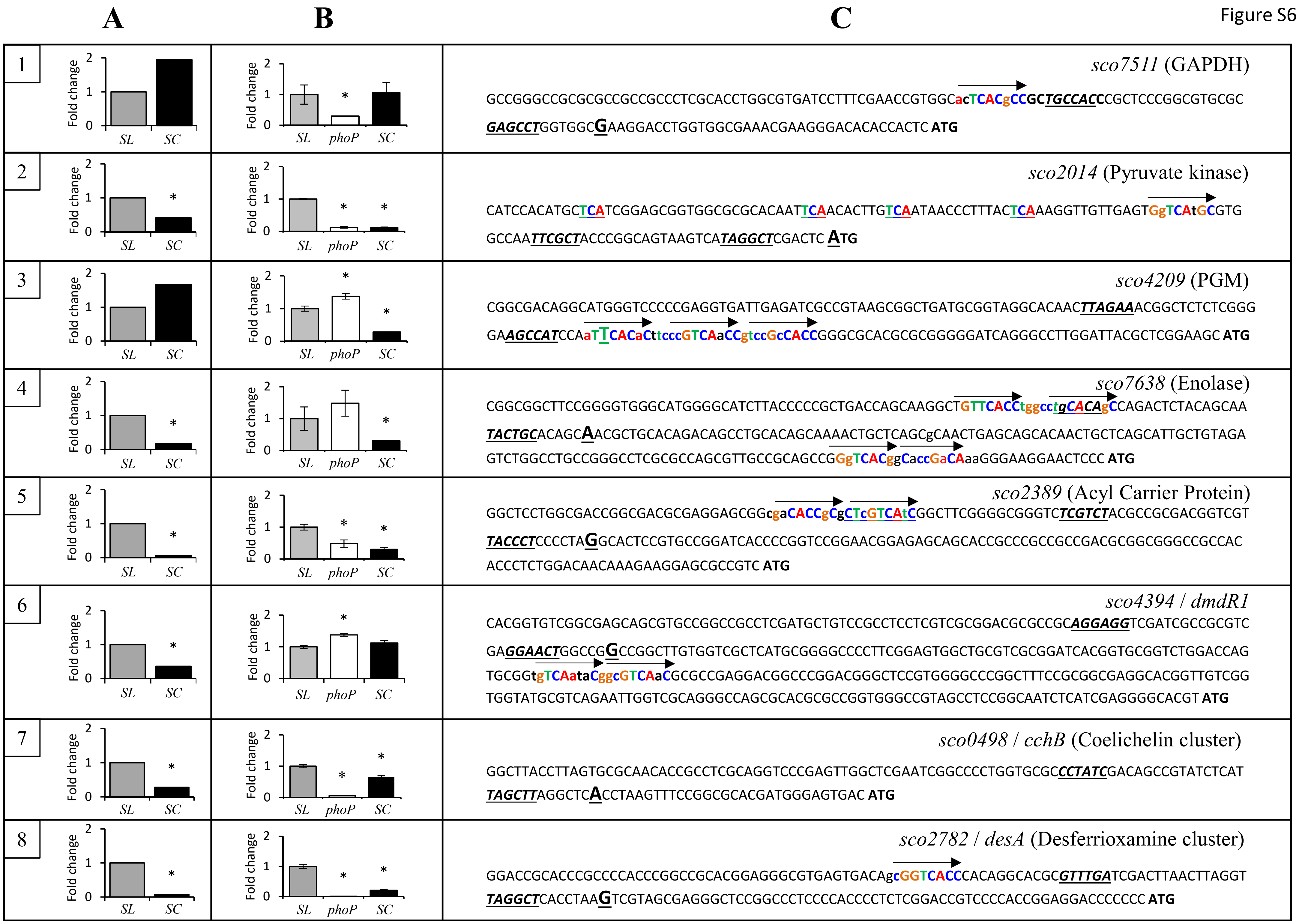
A
B
C
1
2
3
4
5
6
7
8
Fold change
SL
SC
phoP
*
sco7511 (GAPDH)
GCCGGGCCGCGCCGCCGCCCTCGCACCTGGCGTGATCCTTTCGAACCGTGGCacTCACgCCGCTGCCACCCGCTCCCGGCGTGCGC
GAGCCTGGTGGCGAAGGACCTGGTGGCGAAACGAAGGGACACACCACTC ATG
sco2014 (Pyruvate kinase)
CATCCACATGCTCATCGGAGCGGTGGCGCGCACAATTCAACACTTGTCAATAACCCTTTACTCAAAGGTTGTTGAGTGgTCAtGCGTG
GCCAATTCGCTACCCGGCAGTAAGTCATAGGCTCGACTC ATG
sco4209 (PGM)
CGGCGACAGGCATGGGTCCCCCGAGGTGATTGAGATCGCCGTAAGCGGCTGATGCGGTAGGCACAACTTAGAAACGGCTCTCTCGGG
GAAGCCATCCAaTTCACaCttcccGTCAaCCgtccGcCACCGGGCGCACGCGCGGGGGATCAGGGCCTTGGATTACGCTCGGAAGC ATG
sco7638 (Enolase)
CGGCGGCTTCCGGGGTGGGCATGGGGCATCTTACCCCCGCTGACCAGCAAGGCTGTTCACCtggcctgCACAgCCAGACTCTACAGCAA
TACTGCACAGCAACGCTGCACAGACAGCCTGCACAGCAAAACTGCTCAGCgCAACTGAGCAGCACAACTGCTCAGCATTGCTGTAGA
GTCTGGCCTGCCGGGCCTCGCGCCAGCGTTGCCGCAGCCGGgTCACggCaccGaCAaaGGGAAGGAACTCCC ATG
sco2389 (Acyl Carrier Protein)
GGCTCCTGGCGACCGGCGACGCGAGGAGCGGcgaCACCgCgCTcGTCAtCGGCTTCGGGGCGGGTCTCGTCTACGCCGCGACGGTCGT
TACCCTCCCCTAGGCACTCCGTGCCGGATCACCCCGGTCCGGAACGGAGAGCAGCACCGCCCGCCGCCGACGCGGCGGGCCGCCAC
ACCCTCTGGACAACAAAGAAGGAGCGCCGTC ATG
sco4394 / dmdR1
CACGGTGTCGGCGAGCAGCGTGCCGGCCGCCTCGATGCTGTCCGCCTCCTCGTCGCGGACGCGCCGCAGGAGGTCGATCGCCGCGTC
GAGGAACTGGCCGGCCGGCTTGTGGTCGCTCATGCGGGGCCCCTTCGGAGTGGCTGCGTCGCGGATCACGGTGCGGTCTGGACCAG
TGCGGtgTCAataCggcGTCAaCGCGCCGAGGACGGCCCGGACGGGCTCCGTGGGGCCCGGCTTTCCGCGGCGAGGCACGGTTGTCGG
TGGTATGCGTCAGAATTGGTCGCAGGGCCAGCGCACGCGCCGGTGGGCCGTAGCCTCCGGCAATCTCATCGAGGGGCACGT ATG
sco0498 / cchB (Coelichelin cluster)
GGCTTACCTTAGTGCGCAACACCGCCTCGCAGGTCCCGAGTTGGCTCGAATCGGCCCCTGGTGCGCCCTATCGACAGCCGTATCTCAT
TAGCTTAGGCTCACCTAAGTTTCCGGCGCACGATGGGAGTGAC ATG
sco2782 / desA (Desferrioxamine cluster)
GGACCGCACCCGCCCCACCCGGCCGCACGGAGGGCGTGAGTGACAgcGGTCACCCACAGGCACGCGTTTGATCGACTTAACTTAGGT
TAGGCTCACCTAAGTCGTAGCGAGGGCTCCGGCCCTCCCCACCCCTCTCGGACCGTCCCCACCGGAGGACCCCCCC ATG

Figure S6: Differential protein and transcript abundance of putative novel PhoP targets belonging to carbon (*sco7511*/GAPDH, *sco4209*/PGM, *sco2014*/PK, *sco7638*/ENO and *sco2389*/ACP) or iron metabolism (*dmdR1/sco4394*, (*desA/sco2782* and *cchB /sco0498*). Differential protein abundance between *SL* and *SC*. Asterisks signal significant abundance change (ANOVA, adjusted p value < 0.01) in comparison with *SL* taken as reference equal to 1. (**A**) Differential transcript abundance in qRT-pCR between *SL,* its *phoP* mutant and *SC*. Asterisks signal significant abundance change (Student test, adjusted p value < 0.05) in comparison with *SL* taken as reference equal to 1. (**B**) Sequence of the promoter regions of putative novel PhoP regulatory targets from 100nt upstream of the putative -35 sequence to the translational start codon ATG. The transcriptionnal start site is represented by big and bold letter and underlined. Putative -10 and -35 promoter regions are in bold italic and are underlined. Putative PhoP boxes are in colored letters and represented by arrows above the sequence line. Majuscule and small letters of the putative Pho box stand for the most and less conserved bases, respectively, of the consensus proposed by Allenby *et al*. (**C**) Strains were grown for 36 h or 40h for protein and RNA preparations, respectively, on solid R2YE medium, limited in phosphate (1mM) and with glucose (50mM) as main carbon source, at 28°C.

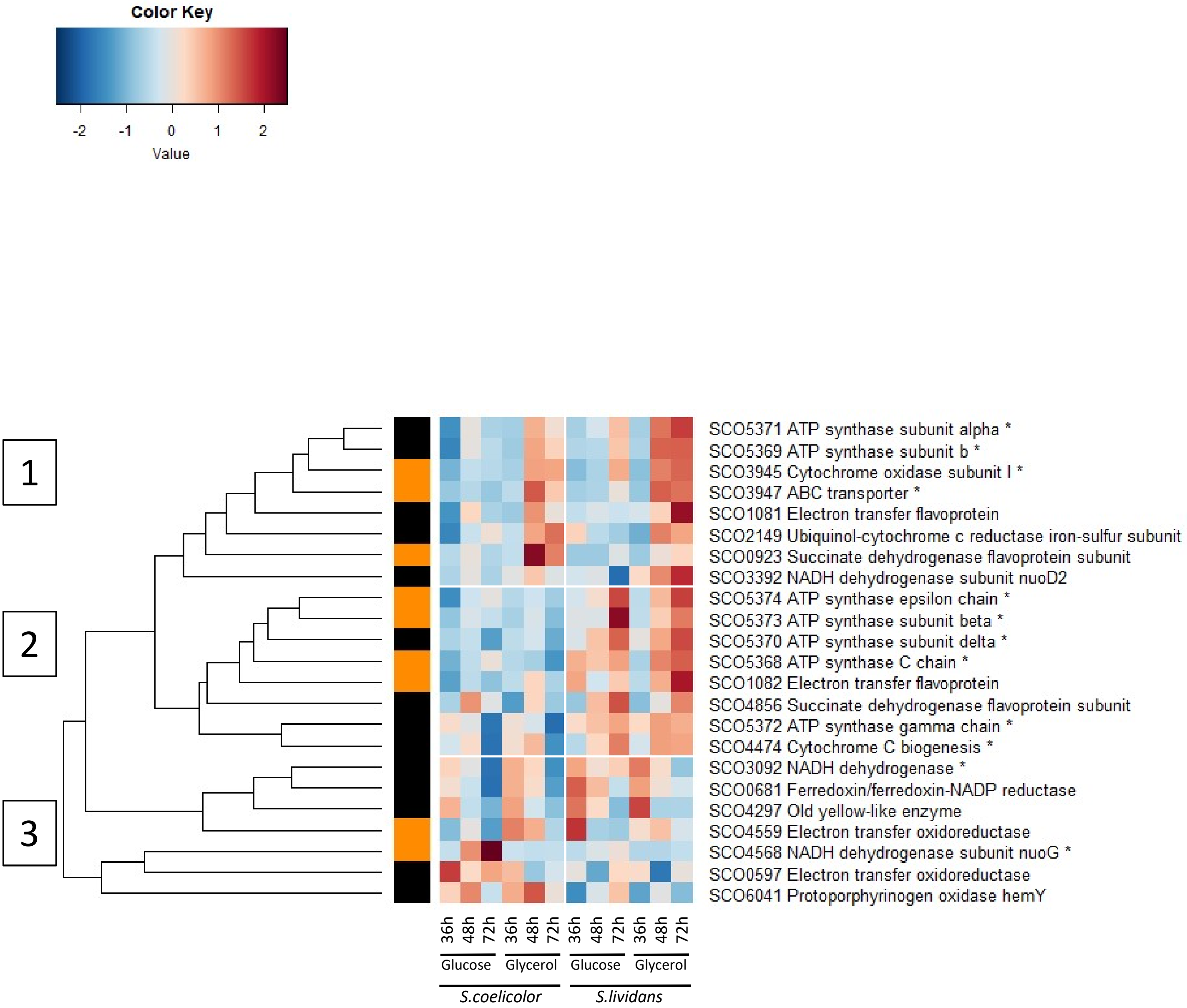

Figure S7: Heatmap representation of enzymes belonging to energetic metabolism (clusters 1 to 3) with significant abundance change (ANOVA, adjusted p value < 0.01) in *S. coelicolor* and *S. lividans* grown on R2YE medium limited in phosphate (1mM) with either glucose or glycerol as main carbon source, for 36, 48 and 72 h at 28°C. Enzymes belonging to the Rex regulon are indicated by an asterisk. Protein identifiers are indicated as SCO numbers for both strains and by predicted functions. The quantification methods are displayed in the vertical bar indicating proteins quantified by SC (orange) or by XIC (black).

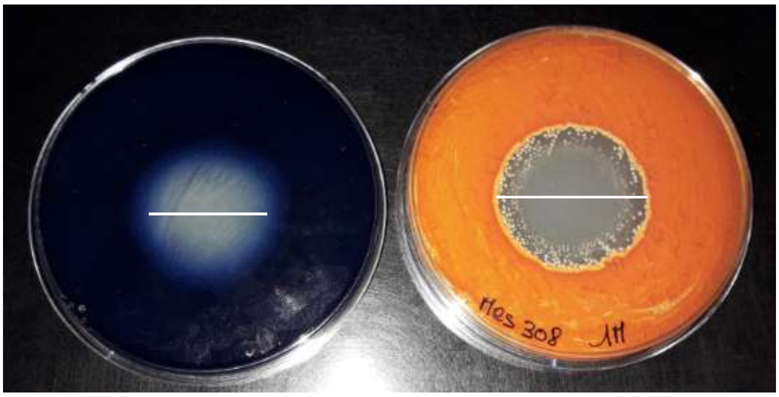

Figure S8: Pictures of growth inhibition zones of 72h grown mycelial lawns of *S. coelicolor* M145 and *S. coelicolor* M1148 deleted for the ACT biosynthetic cluster on which 5µl of the thiol oxydant diamine (1 M) was deposited just after spores plating.

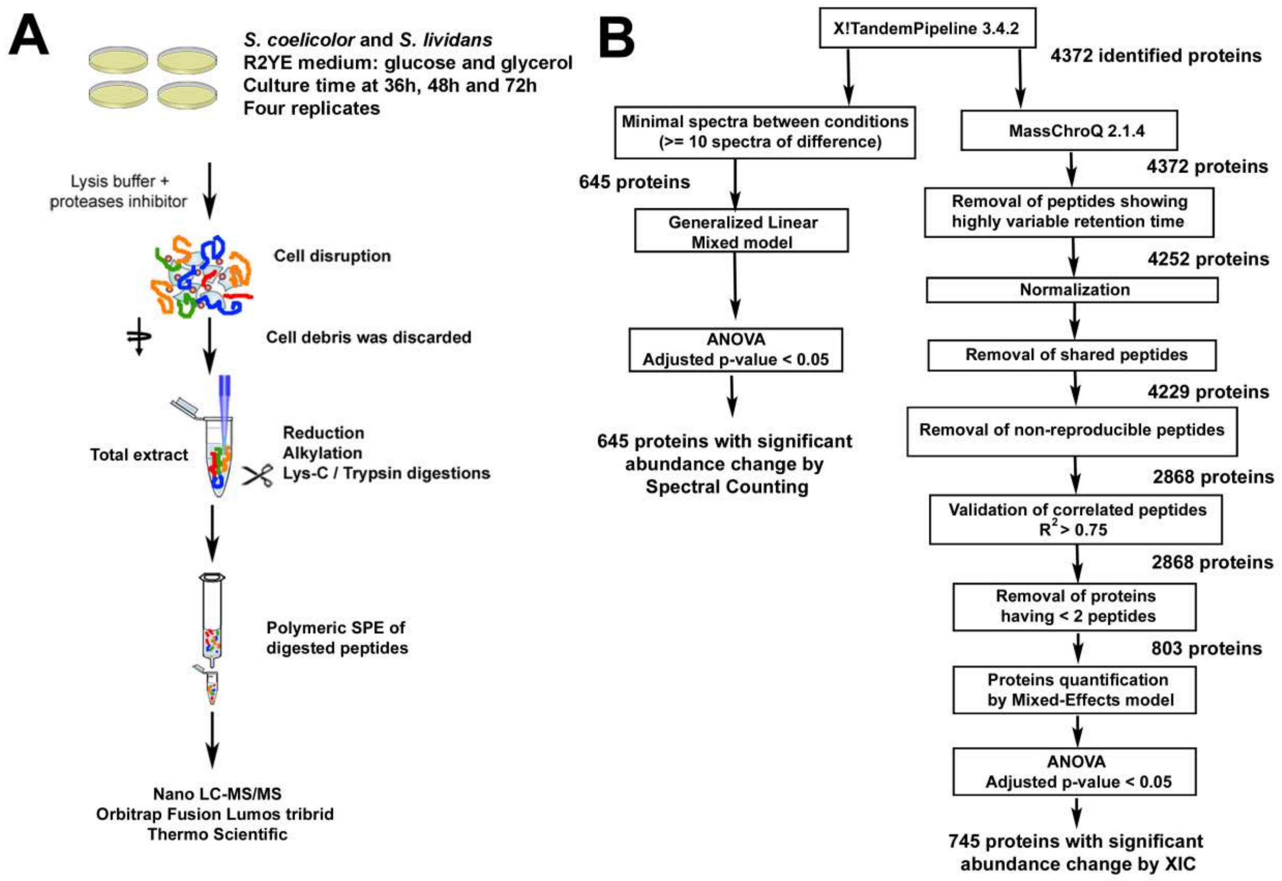

Figure S9: Proteomic workflow. Sample preparation for quantitative proteomics (**A**). Bioinformatic and statistical analysis of high-throughput proteomic data (**B**).

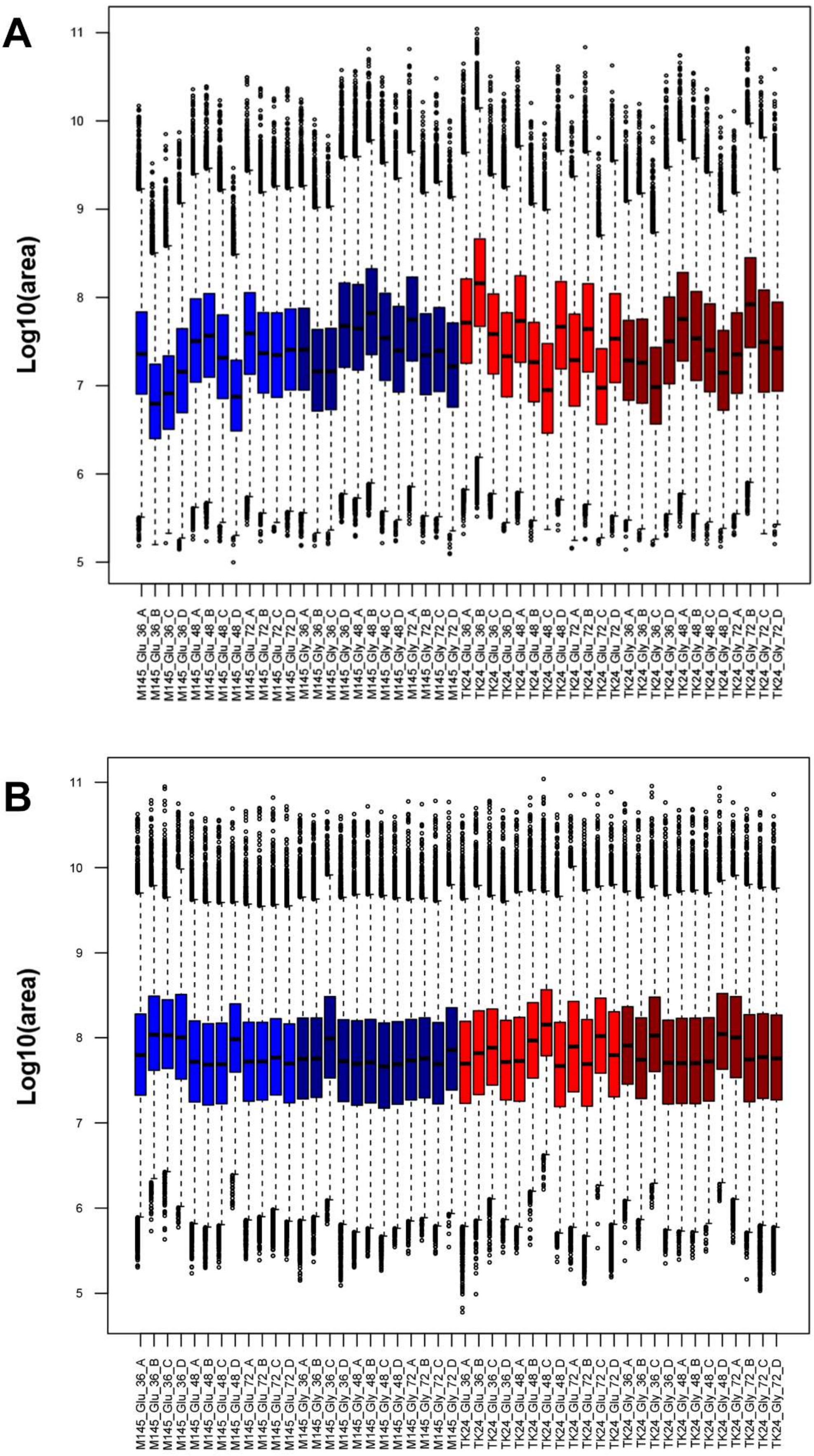

Figure S10: Effect of peptide intensities normalization. LC-MS run samples before (**A**) and after (**B**) peptide intensities normalization.
